# Comparative study of genome-wide plant biomass-degrading CAZymes in white rot, brown rot and soft rot fungi

**DOI:** 10.1080/21501203.2017.1419296

**Published:** 2017-12-24

**Authors:** Ayyappa Kumar Sista Kameshwar, Wensheng Qin

**Affiliations:** Department of Biology, Lakehead University, Thunder Bay, Canada

**Keywords:** Plant biomass, lignocellulose, CAZy, white rot fungi, brown rot fungi, soft rot fungi

## Abstract

We have conducted a genome-level comparative study of basidiomycetes wood-rotting fungi (white, brown and soft rot) to understand the total plant biomass (lignin, cellulose, hemicellulose and pectin) -degrading abilities. We have retrieved the genome-level annotations of well-known 14 white rot fungi, 15 brown rot fungi and 13 soft rot fungi. Based on the previous literature and the annotations obtained from CAZy (carbohydrate-active enzyme) database, we have separated the genome-wide CAZymes of the selected fungi into lignin-, cellulose-, hemicellulose- and pectin-degrading enzymes. Results obtained in our study reveal that white rot fungi, especially *Pleurotus eryngii* and *Pleurotus ostreatus* potentially possess high ligninolytic ability, and soft rot fungi, especially *Botryosphaeria dothidea* and *Fusarium oxysporum* sp., potentially possess high cellulolytic, hemicellulolytic and pectinolytic abilities. The total number of genes encoding for cytochrome P450 monooxygenases and metabolic processes were high in soft and white rot fungi. We have tentatively calculated the overall lignocellulolytic abilities among the selected wood-rotting fungi which suggests that white rot fungi possess higher lignin and soft rot fungi potentially possess higher cellulolytic, hemicellulolytic and pectinolytic abilities. This approach can be applied industrially to efficiently find lignocellulolytic and aromatic compound-degrading fungi based on their genomic abilities.

## 1. Introduction

Naturally, lignocellulose is degraded by a large group of fungi and bacteria (Daniel 2014). Fungi have evolved progressively with their dominant degrading abilities to decay organic debris including plant biomass by penetrating through their hyphae and spores (for long-distance dispersal) (Kendrick 2001). Wood-rotting fungi are categorised into white, brown and soft rot fungi based on their growth substrate preferences and wood-decaying patterns (Kameshwar and Qin 2016a). Moreover, white rot fungi exhibit excellent decaying abilities and are solely responsible for the degradation of lignin and polysaccharides in plant biomass. Microscopy-based studies have differentiated the white rot decay patterns morphologically into (a) simultaneous degradation of lignin and wood polysaccharides (e.g. *Phanerochaete chrysosporium, Trametes versicolor*, (b) selective degradation of plant biomass components (e.g. *Phlebia radiata*) (Rowell and Barbour 1989; Daniel 1994, 2014). However, some fungi like *Heterobasidium annosum* exhibit both simultaneous and selective decay patterns (Daniel 2003).

Brown rot fungi are well characterised as rapid cellulose and hemicellulose degraders, and they access plant polysaccharides by potentially modifying or degrading lignin (Hatakka 2005). These fungi are the major invaders of forest biomass and wood-based constructions. Studies have reported that brown rot fungi have evolved from the saprotrophic white rot fungi by losing several essential genes encoding for lignocellulose-degrading enzymes (Arantes and Goodell 2014). It was reported that hyphae of the brown rot fungi penetrate the cell lumen, colonise the ray cells and axial parenchymal cells to access carbohydrates (Arantes and Goodell 2014).

Most of the ascomycetes and fungi imperfectii cause soft rot decay in the presence of excessive moisture. Soft rot decayed wood exhibits a greyish discolouration and fragmentation which is similar as brown rot. Previous morphological studies have divided the soft rot fungi decay into (a) type-I (where hyphae penetrate secondary cell walls by forming characteristic cavities) and (b) type-II (attacks similarly as ascomycetes and white rot fungi leading to wood cell wall thinning) (Daniel 2014). Wood-decaying fungi and its secreted enzymes are being used commercially in biopulping, kraft pulping (xylanase bleaching), cellulases-based refining, pitch removal (lipases), slime removal (using enzyme cocktail), fibre modification (pulp and paper industries), etc., thus, finding its applications in biodegradation of plant polymers, detoxification and bioremediation of several toxic aromatic compounds and also in bio-based industries (Daniel 2014).

The depolymerising abilities of the wood-rotting fungi are directly proportional to its ability to secrete an array of lignocellulolytic enzymes, aromatic compound and detoxifying enzymes. The plant cell wall-modifying and -degrading enzymes secreted by microorganisms were been classified into six classes by carbohydrate-active enzyme (CAZy) database (Lombard et al. 2013): glycoside hydrolase (GH), glycosyl transferase (GT), auxiliary activity (AA), carbohydrate esterase (CE), polysaccharide lyases (PL) and carbohydrate-binding domains (Lombard et al. 2013). Cellulose, hemicellulose and pectin are the most important and major polysaccharides of the plant cell walls. The presence of lignin (heterophenolic aromatic polymer) along with these components make the plant cell wall recalcitrant (Rytioja et al. 2014). Structurally and functionally plant cell walls are unique, and they can be divided into (a) middle lamella, (b) primary cell wall and (c) secondary cell wall. Chemical composition of plant cell walls varies considerably among monocots, dicots, softwood and hardwood. Primary cell walls of renewable energy crops (monocots, grasses, etc.) contain cellulose and hemicellulose similarly secondary cell walls contains higher amounts of cellulose, varied compositions of hemicellulose and substantial amounts of lignin (Vogel 2008; Rytioja et al. 2014). Where as in dicots primary cell walls contain low xylan, high xyloglucan and mannan, secondary cell walls contain cellulose, hemicellulose and lignin, in dicot plant cell walls pectin is considerably higher (Vogel 2008; Rytioja et al. 2014).

**Figure 1.**
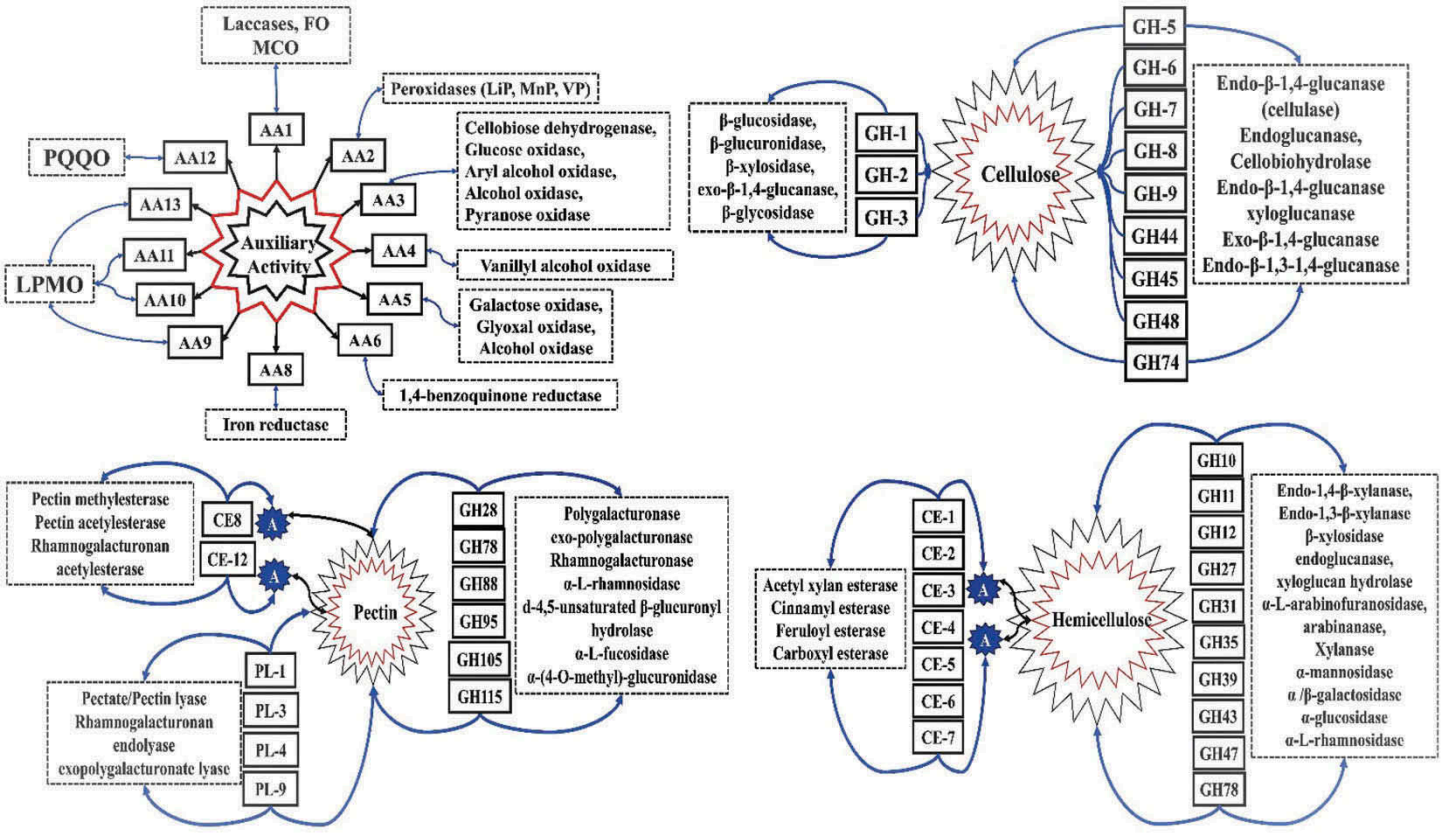
Tentative network of CAZymes involved in depolymerisation of lignin, cellulose, pectin and hemicellulose observed in selected popular white rot, brown rot and soft rot fungi.

Most abundant plant polysaccharide cellulose provides rigidity to the plant cell walls by constituting upto 40–50% of its dry weight. Cellulose is made up of β (1→4) linear chains of d-glucose repeating units linked through hydrogen bonds, wherein the ratio of crystalline to amorphous regions differs between the layers of primary and secondary cell walls and also among the plant species (Harris and Stone 2008; Sjostrom 2013). Hemicellulose constitutes to 20–30% dry weight of the plant biomass; it is mainly composed of xylan (β (1→4) d-xylose units), xyloglucan, β-glucans (β (1→3) (1→4) d-glucose) and mannan (β 1→4 d-mannose); it also contains oligomers of galactose, xylose, arabinose, fucose and glucuronic acid (Sjostrom 2013; Rytioja et al. 2014). The hemicellulose occurs in close association with cellulose, by supporting the microfibrillar structure of cellulose. In plant cell walls, pectin occurs as homogalacturonan (α (1→4) d-galacturonic acid), xylogalacturonan (galacturonan and β (1→3) d-xylose), rhamnogalacturonan-I and rhamnogalacturonan-II. Thus, pectin is the non-cellulosic plant polysaccharide which occurs in intricate associations with other plant cell wall components (Sjostrom 2013; Rytioja et al. 2014). Fungi secrete an array of CAZymes and lignin-degrading enzymes (which includes aromatic compound-degrading and -detoxifying enzymes) for the degradation of lignocellulose (Rytioja et al. 2014) (Figure 1).

Several genomic studies were conducted in the past to reveal the genes encoding for plant biomass-degrading enzymes. In this study, we have selected popular white rot, brown rot and soft rot fungal strains and retrieved their genome-wide annotations to reveal the number of cellulolytic, hemicellulolytic, pectinolytic, lignin-degrading and -detoxifying enzymes (especially cytochrome P450). Present comparative analysis approach can be applied industrially to efficiently find lignocellulose and xenobiotic compound-degrading fungal strains, which can be applied in production of commercially important enzymes and growing biofuel and biorefinery industries.

## 2. Methods

### 2.1. Data retrieval

We have selected (a) 14 popular white rot fungal strains – *Ceriporiopsis subvermispora* B (Fernandez-Fueyo et al. 2012), *Heterobasidion annosum* v2.0 (Olson et al. 2012), *Fomitiporia mediterranea* v1.0 (Floudas et al. 2012), *Phanerochaete carnosa* HHB-10118 (Suzuki et al. 2012), *Pycnoporus cinnabarinus* BRFM 137 (Levasseur et al. 2014), *Phanerochaete chrysosporium* R78 v2.2 (Martinez et al. 2004; Ohm et al. 2014), *Dichomitus squalens* LYAD-421 SS1 (Floudas et al. 2012), *Trametes versicolor* v1.0 (Floudas et al. 2012), *Punctularia strigosozonata* v1.0 (Floudas et al. 2012), *Phlebia brevispora* HHB-7030 SS6 (Binder et al. 2013), *Botrytis cinerea* v1.0 (Amselem et al. 2011), *Pleurotus ostreatus* PC15 v2.0 (Riley et al. 2014; Alfaro et al. 2016; Castanera et al. 2016), *Stereum hirsutum* FP-91666 SS1 v1.0 (Floudas et al. 2012), *Pleurotus eryngii* ATCC90797 (Guillen et al. 1992; Camarero et al. 1999; Ruiz‐Dueñas et al. 1999; Matheny et al. 2006); (b) 15 popular brown rot fungal strains – *Postia placenta* MAD 698-R v1.0 (Martinez et al. 2009), *Fibroporia radiculosa* TFFH 294 (Tang et al. 2012), *Wolfiporia cocos* MD-104 SS10 v1.0 (Floudas et al. 2012), *Dacryopinax primogenitus* DJM 731 SSP1 v1.0 (Floudas et al. 2012), *Daedalea quercina* v1.0 (Nagy et al. 2015), *Laetiporus sulphureus* var v1.0 (Nagy et al. 2015), *Postia placenta* MAD-698-R-SB12 v1.0 (Martinez et al. 2009), *Neolentinus lepideus* v1.0 (Nagy et al. 2015), *Serpula lacrymans* S7.9 v2.0 (Eastwood et al. 2011), *Calocera cornea* v1.0 (Eastwood et al. 2011), *Gloeophyllum trabeum* v1.0 (Floudas et al. 2012), *Fistulina hepatica* v1.0 (Floudas et al. 2015), *Fomitopsis pinicola* FP-58527 SS1 (Floudas et al. 2015), *Hydnomerulius pinastri* v2.0 (Kohler et al. 2015) *and Coniophora puteana* v1.0 (Kohler et al. 2015); (c) 13 popular soft rot fungal strains – *Trichoderma reesei* v 2.0 (Martinez et al. 2008), *Rhizopus oryzae* 99-880 from Broad (Ma et al. 2009), *Aspergillus wentii* v1.0 (De Vries et al. 2017), *Penicillium chrysogenum Wisconsin* 54-1255 (Van Den Berg et al. 2008), *Daldinia eschscholzii* EC12 v1.0, *Hypoxylon* sp. CI-4A v1.0 (Wu et al. 2017), *Aspergillus niger* ATCC 1015 v4.0 (Andersen et al. 2011), *Hypoxylon* sp. EC38 v3.0 (Wu et al. 2017), *Hypoxylon* sp. CO27-5 v1.0 (Wu et al. 2017), *Neurospora crassa* OR74A v2.0 (Galagan et al. 2003), *Lecythophora* sp. AK0013 v1.0 (Damm et al. 2010; U’Ren et al. 2012), *Botryosphaeria dothidea* (Slippers et al. 2004; Desprez-Loustau et al. 2006; Schoch et al. 2006; Slippers and Wingfield 2007; Piškur et al. 2011), *Fusarium oxysporum* sp. lycopersici 4287 v2 (Ma et al. 2010) with available annotated genomes were retrieved from the JGI (Joint Genome Institute) MycoCosm database. Genome-level annotations of the selected fungal strains especially InterPro, eukaryotic orthologous groups (KOG) and CAZy were retrieved from the JGI-MycoCosm database.

### 2.2. Data analysis

Based on the previous literature and the available CAZy annotations, we have classified the genome-wide CAZymes of the above selected white, brown and soft rot fungi. List of CAZymes retrieved from the JGI-MycoCosm database were individually classified into cellulases, hemicellulases and ligninolytic enzymes. We have used Microsoft Excel 2016 to represent the number of genes coding for plant cell wall-degrading enzymes present in the genome-wide annotations of fungi. The images were generated using the option “conditional formatting followed by selecting option colour scales”. Present CAZy database is classified into 145 GHs, 104 GTs, 27 PL, 16 CEs and 13 AA enzymes. We have used the available annotations and literature, to separate the CAZymes into plant cell wall-degrading (cellulolytic, hemicellulolytic, pectinolytic and ligninolytic) enzymes. Similarly, we have analysed the number of protein models encoding for various significant cellular processes using the retrieved KOG and genome-wide InterPro annotations retrieved from JGI-MycoCosm database. We have also calculated the tentative overall lignocellulolytic abilities of white, brown and soft rot fungi based on the genome-wide distribution of lignocellulolytic enzymes in selected fungi. We have performed the hierarchical clustering analysis of the genome-level data (CAZy, InterPro and KOG) of the above selected fungi, using Cluster 3 (De Hoon et al. 2004) and visualised the obtained trees using Java Treeview softwares. Following options were used in Cluster3.0 software: we have uploaded the sample files (containing number of genes encoding for CAZymes, KOG) and selected “hierarchical” clustering, “cluster” options for both the genes and arrays with complete linkage clustering method. The. CDT file obtained from the Cluster3.0 software was imported into the Java Treeview software and the corresponding images were generated and further exported. The hierarchical dendrograms of the plant cell wall-degrading CAZymes, ligninolytic, cellulolytic, hemicellulolytic, pectinolytic and KOG were reported in the Supplementary Material file.

## 3. Results and discussions

Basidiomycetes fungi were highly studied and classified based on their plant biomass decaying abilities into white rot, brown rot and soft rot fungi. White rot fungi are the efficient plant biomass degraders with its specialty lying in degradation of aromatic compounds, thus giving a characteristic white appearance to the decayed wood. Brown rot fungi represent about 6–7% of the basidiomycete fungi. *Phanerochaete chrysosporium* genome was the first basidiomycete complete genome sequence to be published in the year 2004 by Martinez et al. (2004), which has revealed various significant facts about lignocellulose degradation mechanisms (Martinez et al. 2004; Ohm et al. 2014). After this study, the complete genome sequences of several basidiomycetes fungi were revealed in the recent years (Kameshwar and Qin 2016b). Development and advancement of genome repositories such as 1000 fungal genome project and JGI MycoCosm have fastened various findings about the fungal metabolism, physiology and degrading mechanisms (Grigoriev et al. 2013). As mentioned above, we have selected 14 white rot fungi, 15 brown rot fungi and 13 soft rot fungi whose complete annotated genome sequences are published and publicly available. We have retrieved the genome-wide annotations such as InterPro, CAZy, KOG for all the selected fungal strains. Based on the available literature and CAZy architecture, we have separated lignocellulose CAZymes into their respective cellulose-, hemicellulose-, pectin- and lignin-depolymerising enzymes.

**Figure 2.**
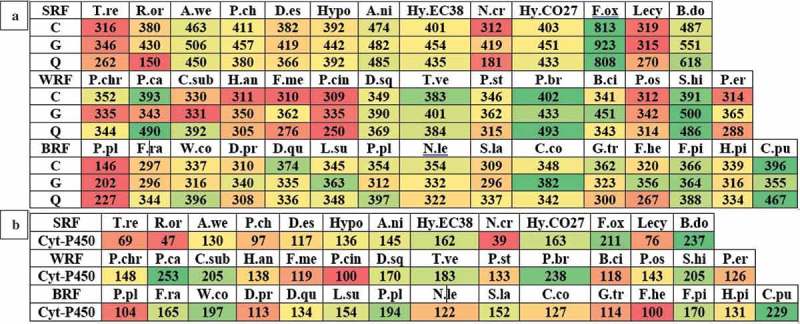
Heatmaps showing the genome-wide distribution of (a) metabolism (C = energy production and conversion, G = carbohydrate transport and metabolism and Q = secondary metabolites biosynthesis, transport and catabolism) and (b) number of cytochrome P450-encoding genes in selected popular white rot, brown rot and soft rot fungi.

The JGI-MycoCosm database classifies and annotates the fungal genomes using the KOG tool (a eukaryotic version of cluster of orthologous groups) that is used for identifying the ortholog and paralog proteins. The KOG groups are divided into four functional groups: (a) cellular processes and signalling, (b) information storage and processing, (c) metabolism and (d) poorly characterised. These four functional groups are further divided into different classes based on their functional characteristics (Table S1). We have retrieved the classified genome KOG groups and their respective function level annotated gene numbers for all the selected wood-rotting fungi. We have observed that *T. reesei, Lecythophora* sp., *P. placenta, F. mediterranea, C. subvermispora* genomes contain lower number of genes classified under the above-mentioned four KOG functional groups. At the same time, higher number of genes encoding for the KOG functional groups were observed among the *F. oxysporum, R. oryzae, P. brevispora, S. hirsutum, C. puteana, F. pinicola* fungi (Figure 2A). In this study, we have specifically compared the total number of genes encoding for KOG functional processes encoding for energy production and conversion (C), carbohydrate transport and metabolism (G) and secondary metabolite biosynthesis and transport (Q). The descending order of fungi based on the number of genes encoding for KOG processes C, G, Q was *F. oxysporum, B. dothidea, P. brevispora, S. hirsutum, C. puteana* and *F. pinicola* respectively. Similarly, lower number of genes were observed among *Lecythophora* sp., *T. reesei, N. crassa* (soft rot), *P. cinnabarinus, P. ostreatus* (white rot) and *P. placenta* MAD-698 R 1.0 respectively (Figure 2B). Total number of genes encoding for different KOG processes of the selected fungi were reported in the supplementary information (Figure S1). Individual distribution of different KOG functional groups among the selected white rot fungi (Table S2), brown rot fungi (Table S3) and soft rot fungi (Table S3) was also reported in the supplementary information.

Several studies have proved the strong involvement of cytochrome P450 monooxygenases in the degradation of aromatic and xenobiotic compounds present in the environment by fungi (Van Gorcom et al. 1998; Črešnar and Petrič 2011). In fungi, cytochrome P450 monooxygenases occur in multiple copies as they play wide range of roles especially in detoxification and degradation (Kameshwar and Qin 2017). Among the selected wood-rotting fungi, *P. carnosa* (253), *P. brevispora* (238), *B. dothidea* (237), *C. puteana* (229), *F. oxysporum* (211) contains higher and *N. crassa* (39), *R. oryzae* (47), *T. reesei* (69), *Lecythophora* sp. (76) lower number of genes encoding for cytochrome P450 monooxygenase-encoding genes (Figure 2B). White rot fungi harbour higher number of genes encoding for cytochrome P450 monooxygenases followed by brown rot fungi and soft rot fungi.

## 4. Distribution of CAZymes among white rot, brown rot and soft rot fungi

**Figure 3.**
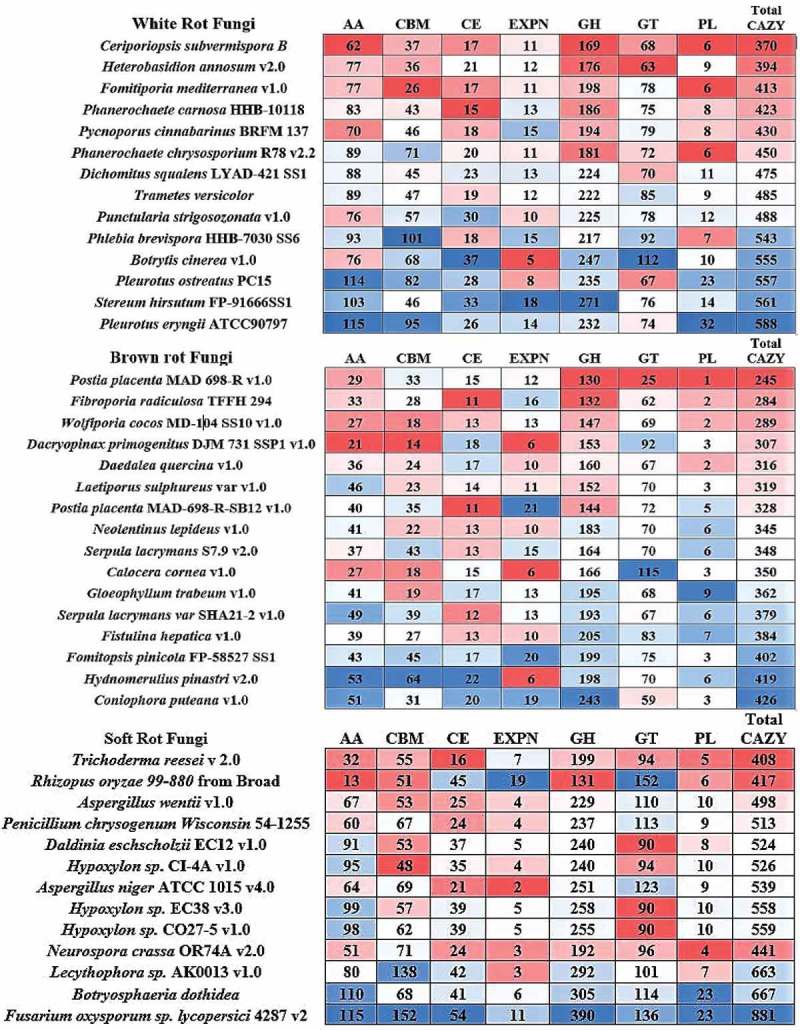
Heatmap showing the genome-wide distribution of CAZymes from selected popular white rot, brown rot and soft rot fungi.

Present-day CAZy database comprises of 145 GHs, 104 GTs, 27 PL, 16 CEs, 13 AA and 81 carbohydrate binding modules (Cantarel et al. 2009; Lombard et al. 2013). Further to this classification, GH classes GH-5, GH-13, GH-30, GH-43 are further divided into 53, 42, 8, 37 subfamilies respectively (Henrissat 1991; Henrissat and Davies 1997). Total number of CAZymes distributed among the selected fungi ranges between 370 (*C. subvermispora*) and 588 (*P. eryngii*) in white rot fungi, 245 (*P. placenta*) and 426 (*C. puteana*) in brown rot fungi, and 408 (*T. reesei*) and 881 (*F. oxysporum*) in soft rot fungi respectively (Figure 1). On average, the total number of CAZymes distributed among the selected fungi were 366 in brown rot, 480 in white rot and 553 in soft rot fungi respectively. Genome-wide distribution of CAZymes among the selected white rot, brown rot and soft rot fungi was clearly listed in the heatmaps (Figure 3) (Figure S2).

### 4.1. Lignin-degrading CAZymes

Fungi secrete an array of oxidative enzymes for the degradation of lignin and other various aromatic compounds. The Fungal oxidative lignin enzymes database has classified lignin-degrading enzymes into two major classes: lignin-oxidising enzymes (LO) and lignin-degrading auxiliary enzymes (Levasseur et al. 2008; Kameshwar and Qin 2016a). LO include laccase (LO1), lignin peroxidase (LO2), manganese peroxidase (LO2), versatile peroxidase (LO2) and cellobiose dehydrogenase (CDH; LO3). Similarly, lignin-degrading auxiliary enzymes majorly includes hydrogen peroxide-generating enzymes such as aryl alcohol oxidase (LDA1), vanillyl alcohol oxidase (LDA2), glyoxal oxidase (LDA3), pyranose oxidase (LDA4), galactose oxidase (LDA5), glucose oxidase (LDA6) and benzoquinone reductase (LDA7) (Levasseur et al. 2008; Kameshwar and Qin 2016a).

In CAZy, lignin-degrading enzymes are classified under the AA class enzymes. We have used CAZy database structure and previous literature to determine the genome-wide lignin-degrading CAZymes and ligninolytic capacities of the selected wood-rotting fungi (Levasseur et al. 2013). LO (laccase, peroxidases (LiP, MnP, VP) and CDH) were classified among AA-1, AA-2, AA-3_1 class enzymes Lignin-degrading auxiliary enzymes were classified among AA-3, AA-4, AA-5, AA-6, AA-8 class enzymes . The genome-wide distribution of lignin-degrading AA enzymes among the selected white rot, brown rot and soft rot fungi is listed (Figure S3). These results convey that white rot fungi possess higher number of laccase-encoding genes when compared to brown rot and soft rot fungi (e.g. >10 laccase-encoding genes were observed in *S. hirsutum, H. annosum, P. ostreatus, F. mediterranea, D. squalens, P. strigosozonata, B. cinerea, P. eryngii)*. Genes encoding for ferroxidases were found to be mostly reduced to one to two copies in white and brown rot fungi. However, most of the soft rot fungi possessed two ferroxidases genes and other fungi harboured three to four ferroxidase genes.

Genes encoding for AA2 class enzymes (lignin (LiP), manganese (MnP), versatile (VP) peroxidases) were found to be reduced among brown and soft rot fungi to few copies (0–4 genes). While genomes of white rot fungi harbour more than five gene copies for AA2 class enzymes with higher number (26) of AA2-encoding genes were observed in *T. versicolor*. Total number of genes encoding for glucose methanol choline oxidoreductases or AA class 3 enzymes dominate in number when compared to other lignin-degrading AA enzymes. Especially, the number of genes encoding for aryl alcohol oxidase and glucose-1-oxidase (AA-3_2 subclass) outnumbers other AA-class enzymes. Higher number of AA-3 class enzymes were observed in white rot fungi (48 in *S. hirsutum*) followed by soft rot fungi (46 in *B. dothidea*) and brown rot fungi (30 in *L. sulphureus*) respectively. In most of the white and brown rot fungi, genes encoding for vanillyl alcohol oxidase (AA4) were reduced to 0, with some fungi comprises around one to three gene sequences. Except in *T. reesei, R. oryzae* and *N. crassa,* all the selected soft rot fungi possessed the gene sequences coding for vanillyl alcohol oxidase. Genes encoding for AA5 class enzymes (galactose, glyoxal and alcohol oxidase), AA6 (benzoquinone reductases) were comparatively higher among the white rot fungi. These results convey that white rot fungi harbour multiple gene copies encoding for lignin-degrading and its auxiliary enzymes when compared to brown and soft rot fungi (Figure 3). Finally, the number of genes encoding for lytic polysaccharide monooxygenases (LPMO) classified under AA-9, AA-10, AA-11 and AA-13 class enzymes were higher in number among the white rot fungi followed by the soft rot fungi respectively (Figure 3). Based on the total number of genes encoding for lignin-degrading AA enzymes, we propose that ligninolytic ability of white rot fungi is higher followed by soft rot fungi (Figure S3).

Studies have revealed that fungi secrete a wide range of aromatic compound-degrading and -detoxifying enzymes during the process of lignin degradation (Kameshwar and Qin 2017). Large group of enzymes encoding for aromatic ring and epoxide hydroxylases, intra- and extra-dioxygenases, alcohol dehydrogenases (Fentons reagents), iron reductase, ferredoxin, catalase, oxidoreductases, cytochrome P450 monooxygenases and other large set of enzymes were involved in degradation of lignin (Kameshwar and Qin 2017). Previous studies have reported the strong dependence of white rot and brown rot fungi on Fenton’s chemistry in the degradation of lignocellulosic units of plant biomass. Apart from the lignin-degrading AA CAZymes, aromatic compound-degrading, -detoxifying enzymes and highly reactive free radicals such as hydrogen peroxide, hydroxy radicals, superoxide and reactive singlet oxygen ions also play a crucial role in degradation of lignocellulosic units (Figure S2).

### 4.2. Cellulose-degrading cazymes

Fungi mainly secrete three classical enzymes: endoglucanses, exoglucanases and β-glucosidases for the hydrolysis of cellulose (Rytioja et al. 2014). Endoglucanases/β-(1→4)-endoglucanases hydrolases cellulose chains by releasing glucooligosaccharides while cellobiohydrolases (CBH)/exoglucanases liberates cellobiose from end chains of cellulose. CBH are divided into CBHI and CBHII, based on location of the cleavage sites either on reducing or non-reducing ends of the cellulose. β-Glucosidases release individual glucose units from the shorter oligosaccharide chains (Rytioja et al. 2014). We have reported the most commonly expressing cellulase-encoding genes among the selected fungal strains. In most of the selected wood-rotting fungi, especially brown and soft rot fungi, we have commonly observed that, the genes encoding for GH-8, GH-44, GH-48 class enzymes (cellulases) were totally reduced to 0. Similarly, in brown and soft rot fungi, genes encoding for GH-9, GH-45, GH-74 and GH-38 class enzymes were reduced to single copies (Figure S4). In white and brown rot fungi, genes encoding for GH-1, GH-2 class enzymes were found to occur in between the range of one and five gene copies. Most of the soft rot fungal genomes contain two to three gene copies of GH-1 class enzymes and 5–10 gene copies of GH-2 class enzymes respectively. However, among all the cellulolytic GHs, the number of genes encoding for the GH-3, GH-5 class enzymes outnumbered other cellulolytic GH class enzymes among the white, brown and soft rot fungi. Among the selected wood-rotting fungi, the total number of cellulases were higher among *P. eryngii* (69), *P. ostreatus* (68) (white rot fungi), *H. pinastri* (51), *C. puteana* (49) (brown rot fungi) and *F. oxysporum* (73) and *Lecythophora* sp. (63) (soft rot fungi) respectively (Figure 3). Our analysis reports that among all the selected wood-rotting fungi, GH class enzymes GH-1, GH-2, GH-3, GH-5 and GH-7 occurs in higher copy numbers. At the same time, lower number of cellulases were found in *H. annosum* (39), *C. subvermispora* (38) (white rot), *P. placenta* (29), *F. radiculosa* (29) (brown rot) and *N. crassa* (36), *R. oryzae* (26) (soft rot) respectively (Figure S4). Recent studies have reported that along with classical cellulases strong oxidoreductases such as CDH and LPMO also partake in degradation of cellulose. Based on total number of genes encoding for cellulases among the selected white, brown and soft rot fungi, we report that the cellulolytic ability is lower in white rot fungi when compared to brown rot and soft rot fungi. CDH and LPMO enzymes work cooperatively for depolymerising cellulose, as CDH produces highly reactive hydroxy radicals through Fenton’s chemistry which plays a dual role by modifying lignin and providing electrons for LPMO-based cellulose degradation (Rytioja et al. 2014) (Figure S2).

### 4.3. Hemicellulose-degrading CAZymes

Compared to cellulose, the microbial degradation of hemicellulose is performed by a specific set of CAZymes, which is majorly due to its complex structure. Classical enzymes such as β (1→4) endoxylanases, xylobiohydrolase, β (1→4) xylosidases which are involved in hydrolysis of xylan backbone, xylan into xylobiose, releases d-xylose units from xylooligosaccharides and hydrolyses xylobiose units to monomeric units respectively (Ghosh and Nanda 1994; Polizeli et al. 2005; Van Den Brink and De Vries 2011; Rytioja et al. 2014). Studies have reported that cellulases (endoglucanases, CBH and beta glucosidases) are involved in hydrolysis of cellulose like xyloglucan and β-glucan backbone structures (Ghosh and Nanda 1994; Polizeli et al. 2005; Van Den Brink and De Vries 2011). Other enzymes such as β (1→4) endo mannanases and β (1→4) mannosidases cleaves mannan back bone structures by releasing monomeric d-mannose units (Ghosh and Nanda 1994; Polizeli et al. 2005; Van Den Brink and De Vries 2011). Apart from these GH enzymes such as CDHs, LPMO are involve in oxidative cleavage of hemicellulose and CEs are involved in O-de-N-deacylation of acetylated plant cell wall residues, especially hemicellulose, pectin and lignin. In most of the selected white and brown rot fungi, genes encoding for CE classes CE-2, CE-3, CE-5, CE-6, CE-7 and GH classes GH-11, GH-39 were completely reduced between one and two gene copies. Based on the total number of genes coding for hemicellulolytic enzymes, we have calculated the hemicellulolytic ability of the selected wood-rotting fungi. In white rot fungi, total number of hemicellulolytic genes varies in between 31 *C. subvermispora* (low) and 63 *B. cinerea* (high), where as in brown rot fungi number of genes varies between 23 *P. placenta* MAD-698 Rv.1.0 (low) and 52 *C. puteana* (high). Contrastingly, soft rot fungi harbours higher number of genes encoding for hemicellulolytic enzymes varying in between 43 *T. reesei* (low) and 135 *F. oxysporum* (high). These results convey that soft rot fungi exhibit higher hemicellulolytic ability followed by white rot fungi (Figure S5) (Figure S2).

### 4.4. Pectin-degrading cazymes

Pectin (a non-cellulosic polysaccharide present in plant cell walls) majorly comprises of galacturonic acid which is intricately connected with the cellulose and hemicellulose units. Majorly pectin occurs in primary and middle lamella of plant cell walls (Rytioja et al. 2014). Structurally pectin can be classified as simple, e.g. homogalacturonan (linear polymer of α (1→4) d-galacturonic acids, methylated at C-6 and acetylated at C-3 positions), xylogalacturonan (chain of galacturonic acid is connected to β (1→3) d-xylose units) and complex pectin, e.g. rhamnogalacturonan-I and II (which contains glycosyl residues such as 2-O-methyl xylose, 2-O-methyl fucose, acetic acid, 2-keto-3-deoxy-d-lyxo heptulosaric acid and 2-keto-3-deoxy-d-mannooctulosonic acid) (Sjostrom 2013; Rytioja et al. 2014). Wood-rotting fungi secrete an arsenal of enzymes involved in depolymerisation of pectin which includes endo-polygalacturonases, exo-polygalacturonases, xylogalacturonan hydrolases, endo-rhamnogalacturonase, rhamnogalacturonan rhamnohydrolase, pectin and pectate lyases, and rhamnogalacturonan hydrolases. Endo- and exo-polygalacturonases act on starting and terminal ends by cleaving the linear chain of α (1→4)-d-galacturonic acid present in the homogalacturonan and releases d-galacturonic acid. Similarly, enzymes such as xylogalacturonan hydrolases and endo-rhamnogalacturonase, rhamnogalacturonan rhamnohydrolase and α-rhamnosidase are involved in depolymerisation of xylogalacturonan and rhamnogalacturonan respectively (Rytioja et al. 2014). In most of the white rot fungi, genes encoding for polysaccharide lyase classes PL-1, PL-3 and PL-9 were completely reduced to 0. The number of pectinolytic enzyme-encoding genes varied between 10 (*P. carnosa*) and 49 (*B. cinerea*). Genes encoding for the polysaccharide lyase (PL-1, PL-3, PL-4 and PL-9) and CE class-12 were completely reduced to 0 or 1, in all the selected brown rot fungi. Compared to white rot and brown rot fungi, total number of pectinolytic enzyme-encoding genes varies in between 11 in *T. reesei* (low) and 24 *F. hepatica* (high). Interestingly in *R. oryzae*, genes encoding for pectinolytic enzymes were completely reduced to 0 (except GH-28(18) and CE-8 (6)) (Figure S6). These results convey that tentative overall pectinolytic ability of the soft rot fungi is higher than the selected white rot and brown rot fungi (Figure S2).

### 4.5. Total lignocellulolytic abilities of selected fungi

**Figure 4.**
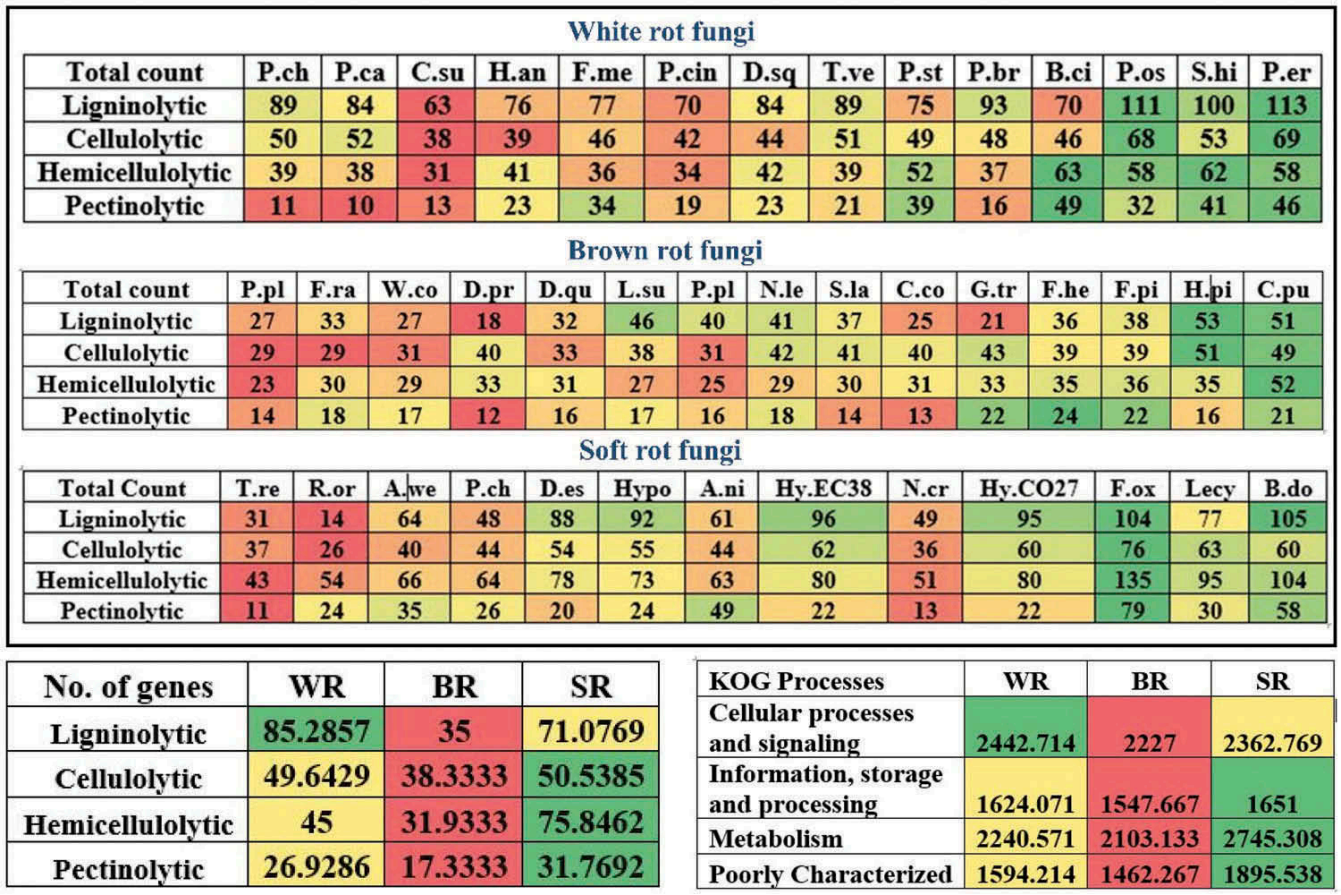
Heatmaps showing the genome-wide distribution of total ligninolytic, cellulolytic, hemicellulolytic and pectinolytic CAZymes in selected popular white rot, brown rot and soft rot fungi and tentative overall.

The total number of lignin-, cellulose-, hemicellulose- and pectin-degrading CAZymes in all the selected white rot, brown rot and soft rot fungi were separated. We have tentatively calculated the total ligninolytic, cellulolytic, hemicellulolytic and pectinolytic abilities by taking average of all the lignocellulolytic CAZymes individually. These results have revealed that, highest ligninolytic ability was observed in *P. eryngii, P. ostreatus, S. hirsutum* (white rot fungi), *H. pinastri, C. puteana* (brown rot fungi) and *B. dothidea, F. oxysporum* (soft rot fungi) respectively. Similarly, the highest cellulolytic ability was observed in *P. eryngii, P. ostreatus* (white rot fungi), *H. pinastri, C. puteana* (brown rot) and *F. oxysporum, Lecythophora* sp. (soft rot fungi) respectively. Highest hemicellulolytic ability was observed in *B. cinerea, S. hirsutum* (white rot fungi), *C. puteana, F. pinicola* (brown rot fungi) and *B. dothidea, F. oxysporum* (soft rot fungi) respectively. Finally, highest pectinolytic ability was observed in *B. cinerea, P. eryngii* (white rot fungi), *F. hepatica, F. pinicola, G. trabeum* (brown rot fungi) and *B. dothidea, F. oxysporum* (soft rot fungi) respectively (Figure 4). We have averaged the total number of genes encoding for lignocellulolytic enzymes to tentatively, to find the overall highest lignocellulolytic abilities. Results obtained from this analysis suggests that white rot fungi (85) possess highest ligninolytic capacity followed by soft rot fungi (71). At the same time, soft rot fungi exhibited potentially highest cellulolytic, hemicellulolytic and pectinolytic abilities by harbouring higher number of genes (Figure 4). Interestingly, the total average of genes encoding for various enzymes involved in cellular processes and signalling were higher in white rot fungi followed by soft rot fungi. Whereas genes encoding for information storage and processing, metabolism processes were higher in soft rot fungi followed by white rot fungi respectively (Figure 4) (Figure S2).

## 5. Conclusion

Degradation of plant biomass by fungi is a highly researched subject for several years. Fungi are the most efficient degraders of the plant biomass (most abundant carbon source on the earth’s surface) and natural scavengers in the environment, thus playing a key role in maintenance of the global carbon cycle. Enzyme systems secreted by fungi are commercially applied in various industries such as paper, pulp, detergents, textile, wine industries and especially fungi are highly studied and applied in the growing biofuel, biorefinery and bioremediation industries (Mäkelä et al. 2014). The decaying ability of the wood-rotting fungi is directly proportional to its ability to secrete plant biomass-degrading enzymes. In this study, we have performed a comparative analysis to understand the genome-wide distribution of lignocellulolytic CAZymes among well-known 14 white rot, 15 brown rot and 13 soft rot fungi. We have separated and classified genome-wide wood-rotting fungal CAZymes into lignin-, cellulose-, hemicellulose- and pectin-degrading enzymes. The total number of genes encoding for ligninolytic, cellulolytic, hemicellulolytic and pectinolytic enzymes calculated in this study reveals that white rot fungi are well equipped with efficient enzyme machinery for the degradation of lignin. The total ligninolytic abilities of white rot fungi (lignin-degrading AA enzymes and cytochrome P450 monooxygenases) were significantly higher than those of soft rot fungi and brown rot fungi. In contrast, total cellulolytic, hemicellulolytic and pectinolytic abilities were highest in soft rot fungi followed by white rot and brown rot fungi. These results suggest that white rot fungal strains are highly suitable for the degradation of lignin, other aromatic compounds and environmental pollutants, whereas soft rot fungal strains are highly suitable in cellulose, hemicellulose and pectin degradation studies, thus highly suitable in biofuel and biorefining industries. We understand that the number of protein-encoding (lignocellulolytic enzymes) genes do not totally determine the complete lignocellulolytic capacity of the fungi as the expression and turnover of these lignocellulolytic enzymes is dependent on various factors and enzymes. However, this study provides preliminary genomic details which are enough to decide on a strain which is comparatively better from the other strains. We believe that understanding the genetic material coding for the lignocellulolytic enzymes will significantly benefit researchers to choose genetically better strain for their studies. However, further relevant studies must be conducted to optimise the appropriate growth and environmental conditions to enhance the expression and protein turnover of these lignocellulolytic enzymes.
