# Supplementary material for: Comparative study of genome-wide plant biomass-degrading CAZymes in white rot, brown rot and soft rot fungi
Source: Mycology. 2017 Dec 24;9(2):93–105. doi: 10.1080/21501203.2017.1419296 (PMC6059041; doi:10.1080/21501203.2017.1419296)
Supplement: Supplementary_Information.pdf [file TMYC_A_1419296_SM6664.pdf]

Comparative Study of Genome-Wide Plant Biomass Degrading CAZymes in White Rot, Brown Rot and Soft Rot Fungi

*Ayyappa Kumar Sista Kameshwar, Wensheng Qin**
Department of Biology, Lakehead University, 955 Oliver Road, Thunder Bay, Ontario, P7B 5E1, Canada.
Wensheng Qin * Email: wqin@lakeheadu.ca, Tel: 807-343-8467

Soft rot fungi	T.re	R.or	A.we	P.ch	D.es	Hypo	A.ni	Hy.EC38	N.cr	Hy.CO27	F.ox	Lecy	B.do
Cellular processes and signaling	1940	3137	2053	2147	2051	2103	2067	2122	2535	2119	4387	1502	2553
Information, storage, processing	1406	2221	1367	1473	1474	1514	1335	1508	1939	1509	2871	1112	1734
Metabolism	2074	2475	2894	2706	2386	2466	2906	2589	2156	2607	5216	1986	3228
Poorly Characterized	1413	2439	1825	1929	1623	1710	1832	1741	1581	1759	3381	1353	2056

White rot Fungi	P.chr	P.ca	C.sub	H.an	F.me	P.cin	D.sq	T.ve	P.st	P.br	B.ci	P.os	S.hi	P.er
Cellular processes and signaling	2423	2284	2300	2347	2291	2227	2318	2576	2406	2855	2335	2314	2868	2654
Information, storage, processing	1713	1539	1482	1641	1457	1553	1575	1725	1504	1871	1615	1570	1791	1701
Metabolism	2179	2401	2111	2001	2010	1944	2218	2364	2091	2765	2367	2094	2664	2159
Poorly Characterized	1567	1601	1435	1643	1501	1610	1567	1599	1568	1792	1658	1496	1715	1567

Brown rot Fungi	P.pl	F.ra	W.co	D.pr	D.qu	L.su	P.pl	N.le	S.la	C.co	G.tr	F.he	F.pi	H.pi	C.pu
Cellular processes and signaling	1590	1976	2330	2048	2244	2264	2426	2262	2083	2501	2202	2147	2421	2456	2455
Information, storage, processing	1035	1329	1649	1353	1598	1648	1563	1566	1526	1759	1579	1482	1753	1711	1664
Metabolism	1150	1976	2296	1960	2197	2282	2231	2153	2051	2192	2129	1995	2338	2124	2473
Poorly Characterized	904	1293	1518	1369	1482	1470	1494	1569	1400	1548	1595	1401	1564	1691	1636

Figure S1: Heatmaps showing the genome wide distribution of KOG's (eukaryotic orthologous groups) in selected popular white rot, brown rot and soft rot fungi.

Table S1: Lists the JGI-MycoCosm KOG group structure and the respective KOG functions divided under the respective KOG groups

KOG Groups	Function Description and KOG ID
**Cellular processes and signaling**	Cell wall/membrane/envelope biogenesis (**M**)<br>Cell motility (**N**)<br>Posttranslational modification, protein turnover, chaperones (**O**)<br>Signal transduction mechanisms (**T**)<br>Intracellular trafficking, secretion, and vesicular transport (**U**)<br>Defense mechanisms (**V**)<br>Extracellular structures (**W**)<br>Nuclear structure (**Y**)<br>Cytoskeleton (**Z**)
**Information storage and processing**	RNA processing and modification (**A**)<br>Chromatin structure and dynamics (**B**)<br>Translation, ribosomal structure and biogenesis (**J**)<br>Transcription (**K**)<br>Replication, recombination and repair (**L**)
**Metabolism**	Energy production and conversion (**C**)<br>Cell cycle control, cell division, chromosome partitioning (**D**)<br>Amino acid transport and metabolism (**E**)<br>Nucleotide transport and metabolism (**F**)<br>Carbohydrate transport and metabolism (**G**)<br>Coenzyme transport and metabolism (**H**)<br>Lipid transport and metabolism (**I**)<br>Inorganic ion transport and metabolism (**P**)<br>Secondary metabolites biosynthesis, transport and catabolism (**Q**)
**Poorly characterized**	General function prediction only (**R**)<br>Function unknown (**S**)

Table S2: Heatmaps showing the genome wide distribution of KOG's (eukaryotic orthologous groups) in selected popular white rot fungi.

**CPS**	**P.chr**	**P.ca**	**C.sub**	**H.an**	**F.me**	**P.cin**	**D.sq**	**T.ve**	**P.st**	**P.br**	**B.ci**	**P.os**	**S.hi**	**P.er**
**M**	76	95	86	100	87	85	87	93	88	117	148	78	135	84
**N**	5	5	5	7	4	6	5	5	6	9	7	6	5	6
**O**	713	700	661	633	671	640	738	765	663	850	663	651	777	705
**T**	705	652	703	703	657	594	658	731	763	822	601	662	745	740
**U**	340	319	299	341	332	328	316	335	313	365	344	337	349	351
**V**	149	132	149	172	139	157	152	197	175	203	158	163	290	218
**W**	91	69	83	104	56	96	73	103	96	107	67	104	188	160
**Y**	102	93	122	84	147	114	92	144	94	148	141	100	167	159
**Z**	242	219	192	203	198	207	197	203	208	234	206	213	212	231
**All**	2423	2284	2300	2347	2291	2227	2318	2576	2406	2855	2335	2314	2868	2654
**ISP**	**P.chr**	**P.ca**	**C.sub**	**H.an**	**F.me**	**P.cin**	**D.sq**	**T.ve**	**P.st**	**P.br**	**B.ci**	**P.os**	**S.hi**	**P.er**
**A**	489	382	385	457	389	402	425	445	392	475	382	381	460	424
**B**	201	184	182	174	168	204	188	236	187	227	186	172	229	190
**J**	366	346	292	327	312	330	338	339	314	388	378	334	344	349
**K**	415	396	379	462	399	377	399	428	383	504	450	439	502	484
**L**	242	231	244	221	189	240	225	277	228	277	219	244	256	254
**All**	1713	1539	1482	1641	1457	1553	1575	1725	1504	1871	1615	1570	1791	1701
**Metabolism**	**P.chr**	**P.ca**	**C.sub**	**H.an**	**F.me**	**P.cin**	**D.sq**	**T.ve**	**P.st**	**P.br**	**B.ci**	**P.os**	**S.hi**	**P.er**
**C**	352	393	330	311	310	309	349	383	346	402	341	312	391	314
**D**	233	219	209	221	215	227	212	247	208	270	199	232	235	271
**E**	258	252	224	226	233	246	246	260	244	312	309	243	297	239
**F**	84	84	77	89	76	71	89	82	81	150	77	82	92	81
**G**	335	343	331	350	362	335	390	401	362	433	451	342	500	365
**H**	98	101	93	95	99	88	101	99	98	132	103	97	119	95
**I**	312	362	302	262	299	281	304	339	286	372	340	302	347	327
**P**	163	157	153	142	140	137	158	169	151	201	204	170	197	179
**Q**	344	490	392	305	276	250	369	384	315	493	343	314	486	288
**All**	2179	2401	2111	2001	2010	1944	2218	2364	2091	2765	2367	2094	2664	2159
**Poorly Characterized**	**P.chr**	**P.ca**	**C.sub**	**H.an**	**F.me**	**P.cin**	**D.sq**	**T.ve**	**P.st**	**P.br**	**B.ci**	**P.os**	**S.hi**	**P.er**
**R**	1137	1208	1039	1207	1053	1238	1136	1186	1129	1324	1248	1073	1291	1110
**S**	430	393	396	436	448	372	431	413	439	468	410	423	424	457
**All**	1567	1601	1435	1643	1501	1610	1567	1599	1568	1792	1658	1496	1715	1567

Table S3: Heatmaps showing the genome wide distribution of KOG's (eukaryotic orthologous groups) in selected popular brown rot fungi.

CPS	P.pl	F.ra	W.co	D.pr	D.qu	L.su	P.pl	N.le	S.la	C.co	G.tr	F.he	F.pi	H.pi	C.pu
M	56	93	94	72	93	101	84	89	101	74	81	92	92	88	92
N	1	3	6	5	6	5	5	6	5	5	5	4	6	5	4
O	316	593	688	605	680	724	689	658	620	656	644	642	724	671	759
T	553	534	651	615	614	589	716	666	576	805	664	597	668	751	652
U	134	308	347	304	333	348	327	338	324	320	332	334	348	345	329
V	204	135	139	145	140	120	187	158	124	198	140	120	150	182	169
W	107	77	83	72	75	55	110	74	63	118	54	66	78	112	111
Y	82	77	115	49	99	106	92	88	85	94	106	103	124	104	136
Z	137	156	207	181	204	216	216	185	185	231	176	189	231	198	203
All	1590	1976	2330	2048	2244	2264	2426	2262	2083	2501	2202	2147	2421	2456	2455
ISP	P.pl	F.ra	W.co	D.pr	D.qu	L.su	P.pl	N.le	S.la	C.co	G.tr	F.he	F.pi	H.pi	C.pu
A	296	354	467	290	445	431	442	395	340	391	407	365	464	425	414
B	103	148	174	154	185	183	161	188	193	226	182	175	210	214	199
J	253	309	351	324	347	361	336	350	346	382	348	340	365	371	355
K	244	326	411	373	390	420	401	403	396	478	399	395	447	442	453
L	139	192	246	212	231	253	223	230	251	282	243	207	267	259	243
All	1035	1329	1649	1353	1598	1648	1563	1566	1526	1759	1579	1482	1753	1711	1664
Metabolism	P.pl	F.ra	W.co	D.pr	D.qu	L.su	P.pl	N.le	S.la	C.co	G.tr	F.he	F.pi	H.pi	C.pu
C	146	297	337	310	374	345	354	354	309	348	362	320	366	339	396
D	129	199	251	202	231	259	219	211	231	254	214	232	251	245	241
E	88	224	267	240	241	269	246	268	238	254	262	226	246	255	264
F	39	70	109	77	82	96	92	96	79	79	91	86	92	86	96
G	202	296	316	340	335	363	312	332	296	382	323	356	364	316	355
H	58	104	119	92	124	113	113	116	105	93	111	105	125	100	127
I	153	311	339	256	313	334	339	310	321	279	326	251	332	303	367
P	108	131	162	135	161	155	159	144	135	161	140	152	174	146	160
Q	227	344	396	308	336	348	397	322	337	342	300	267	388	334	467
All	1150	1976	2296	1960	2197	2282	2231	2153	2051	2192	2129	1995	2338	2124	2473
Poorly	P.pl	F.ra	W.co	D.pr	D.qu	L.su	P.pl	N.le	S.la	C.co	G.tr	F.he	F.pi	H.pi	C.pu
R	700	943	1090	1010	1064	1029	1106	1150	985	1127	1155	998	1138	1167	1190
S	204	350	428	359	418	441	388	419	415	421	440	403	426	524	446
All	904	1293	1518	1369	1482	1470	1494	1569	1400	1548	1595	1401	1564	1691	1636

Table S4: Heatmaps showing the genome wide distribution of KOG's (eukaryotic orthologous groups) in selected popular soft rot fungi.

CPS	T.re	R.or	A.we	P.ch	D.es	Hypo	A.ni	Hy.EC38	N.cr	Hy.CO27	F.ox	Lecy	B.do
M	87	144	110	132	94	88	117	106	93	105	241	90	153
N	5	11	6	6	4	6	5	5	6	5	8	5	6
O	559	859	609	566	627	663	621	656	682	667	1185	523	675
T	516	1012	546	608	514	515	545	524	700	526	1245	370	683
U	313	566	343	338	327	344	310	334	382	336	557	303	324
V	109	93	100	126	129	120	125	133	180	119	287	44	218
W	65	27	40	65	84	73	66	75	118	68	173	11	130
Y	106	86	112	104	99	111	91	96	148	90	258	28	139
Z	180	339	187	202	173	183	187	193	226	203	433	128	225
	1940	3137	2053	2147	2051	2103	2067	2122	2535	2119	4387	1502	2553
ISP	T.re	R.or	A.we	P.ch	D.es	Hypo	A.ni	Hy.EC38	N.cr	Hy.CO27	F.ox	Lecy	B.do
A	346	354	326	360	348	386	312	361	429	347	621	238	441
B	136	277	138	143	174	166	131	176	228	182	380	94	193
J	339	657	347	344	338	344	337	356	383	353	552	325	377
K	393	686	362	388	401	410	360	406	676	414	883	262	484
L	192	247	194	238	213	208	195	209	223	213	435	193	239
	1406	2221	1367	1473	1474	1514	1335	1508	1939	1509	2871	1112	1734
Metabolism	T.re	R.or	A.we	P.ch	D.es	Hypo	A.ni	Hy.EC38	N.cr	Hy.CO27	F.ox	Lecy	B.do
C	316	380	463	411	382	392	474	401	312	403	813	319	487
D	196	358	191	216	201	194	193	198	238	204	415	162	212
E	290	333	428	398	298	312	413	319	281	319	738	291	412
F	95	102	97	103	92	90	98	91	95	94	169	93	111
G	346	430	506	457	419	442	482	454	419	451	923	315	551
H	95	101	133	123	106	110	136	113	115	112	199	109	141
I	297	385	429	399	318	343	428	370	277	372	695	288	458
P	177	236	197	219	204	191	197	208	238	219	456	139	238
Q	262	150	450	380	366	392	485	435	181	433	808	270	618
	2074	2475	2894	2706	2386	2466	2906	2589	2156	2607	5216	1986	3228
Poorly	T.re	R.or	A.we	P.ch	D.es	Hypo	A.ni	Hy.EC38	N.cr	Hy.CO27	F.ox	Lecy	B.do
R	1030	1907	1452	1557	1216	1307	1459	1321	1136	1343	2763	1009	1649
S	383	532	373	372	407	403	373	420	445	416	618	344	407
	1413	2439	1825	1929	1623	1710	1832	1741	1581	1759	3381	1353	2056

Figure-S2: Hierarchical dendrograms of all the selected fungi based on their genome wide distribution of plant cell wall degrading enzymes:

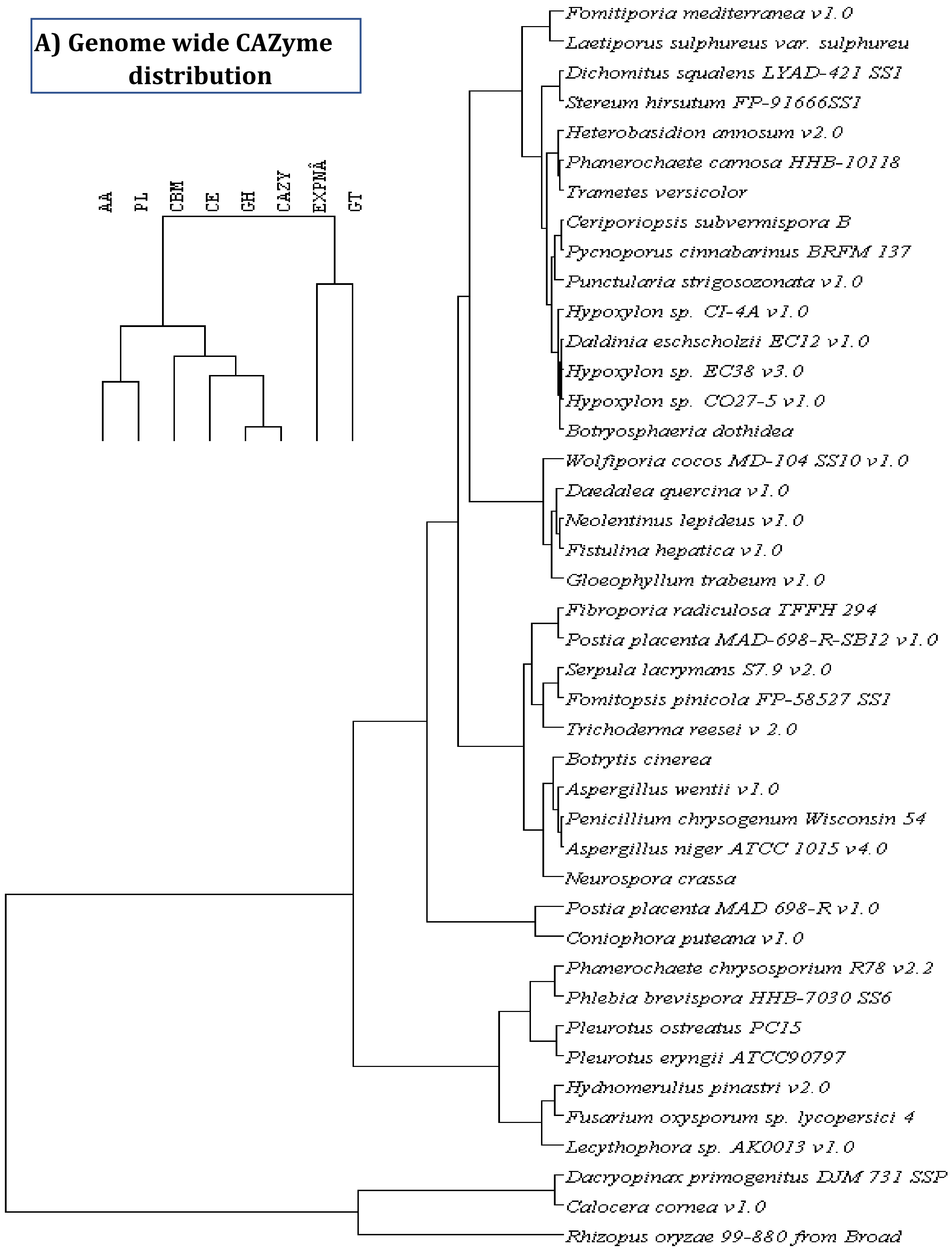

B) Genome wide Auxiliary Activity (Ligninolytic) enzymes

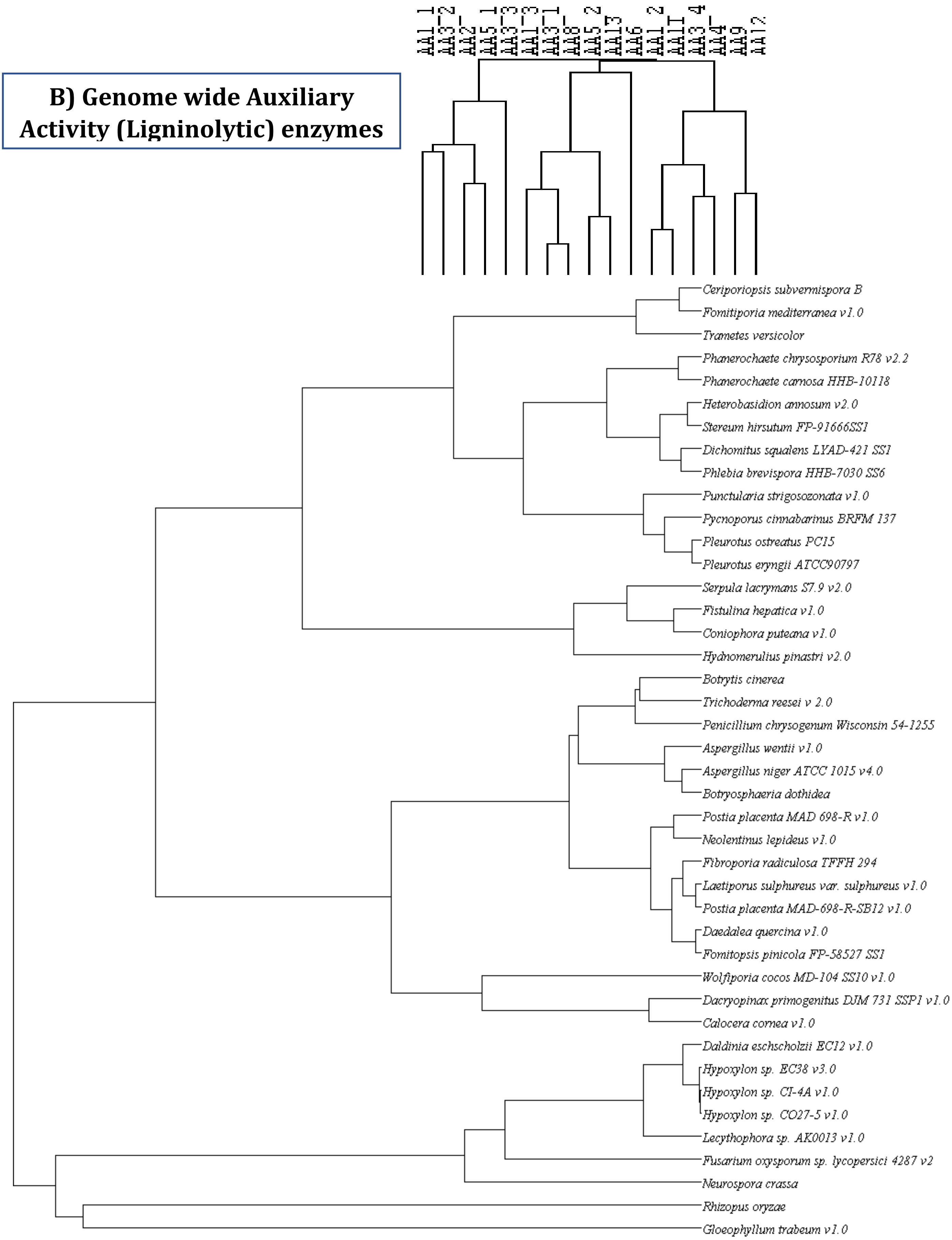

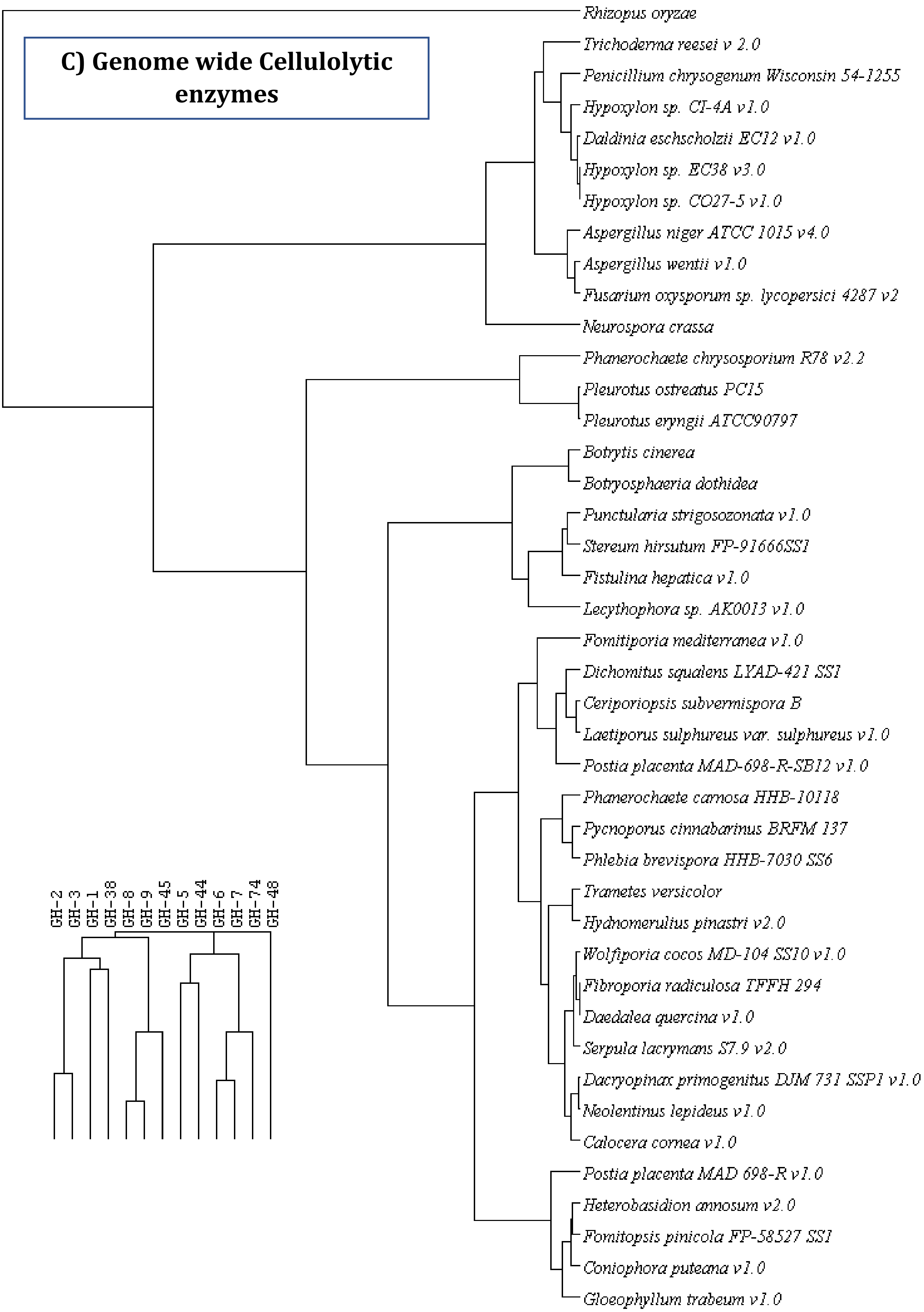
C) Genome wide Cellulolytic enzymes
Rhizopus oryzae
Trichoderma reesei v 2.0
Penicillium chrysogenum Wisconsin 54-1255
Hypoxylon sp. CI-4A v1.0
Daldinia eschscholzii EC12 v1.0
Hypoxylon sp. EC38 v3.0
Hypoxylon sp. CO27-5 v1.0
Aspergillus niger ATCC 1015 v4.0
Aspergillus wentii v1.0
Fusarium oxysporum sp. lycopersici 4287 v2
Neurospora crassa
Phanerochaete chrysosporium R78 v2.2
Pleurotus ostreatus PC15
Pleurotus eryngii ATCC90797
Botrytis cinerea
Botryosphaeria dothidea
Punctularia strigosozonata v1.0
Stereum hirsutum FP-91666SS1
Fistulina hepatica v1.0
Lecythophora sp. AK0013 v1.0
Fomitiporia mediterranea v1.0
Dichomitus squalens LYAD-421 SS1
Ceriporiopsis subvermispora B
Laetiporus sulphureus var. sulphureus v1.0
Postia placenta MAD-698-R-SB12 v1.0
Phanerochaete carnosa HHB-10118
Pycnoporus cinnabarinus BRFM 137
Phlebia brevispora HHB-7030 SS6
Trametes versicolor
Hydnomerulius pinastri v2.0
Wolfiporia cocos MD-104 SS10 v1.0
Fibroporia radiculosa TFFH 294
Daedalea quercina v1.0
Serpula lacrymans S7.9 v2.0
Dacryopinax primogenitus DJM 731 SSP1 v1.0
Neolentinus lepideus v1.0
Calocera cornea v1.0
Postia placenta MAD 698-R v1.0
Heterobasidion annosum v2.0
Fomitopsis pinicola FP-58527 SS1
Coniophora puteana v1.0
Gloeophyllum trabeum v1.0
GH-2
GH-3
GH-1
GH-38
GH-8
GH-9
GH-45
GH-5
GH-44
GH-6
GH-7
GH-74
GH-48

D) Genome wide Hemicellulolytic enzymes

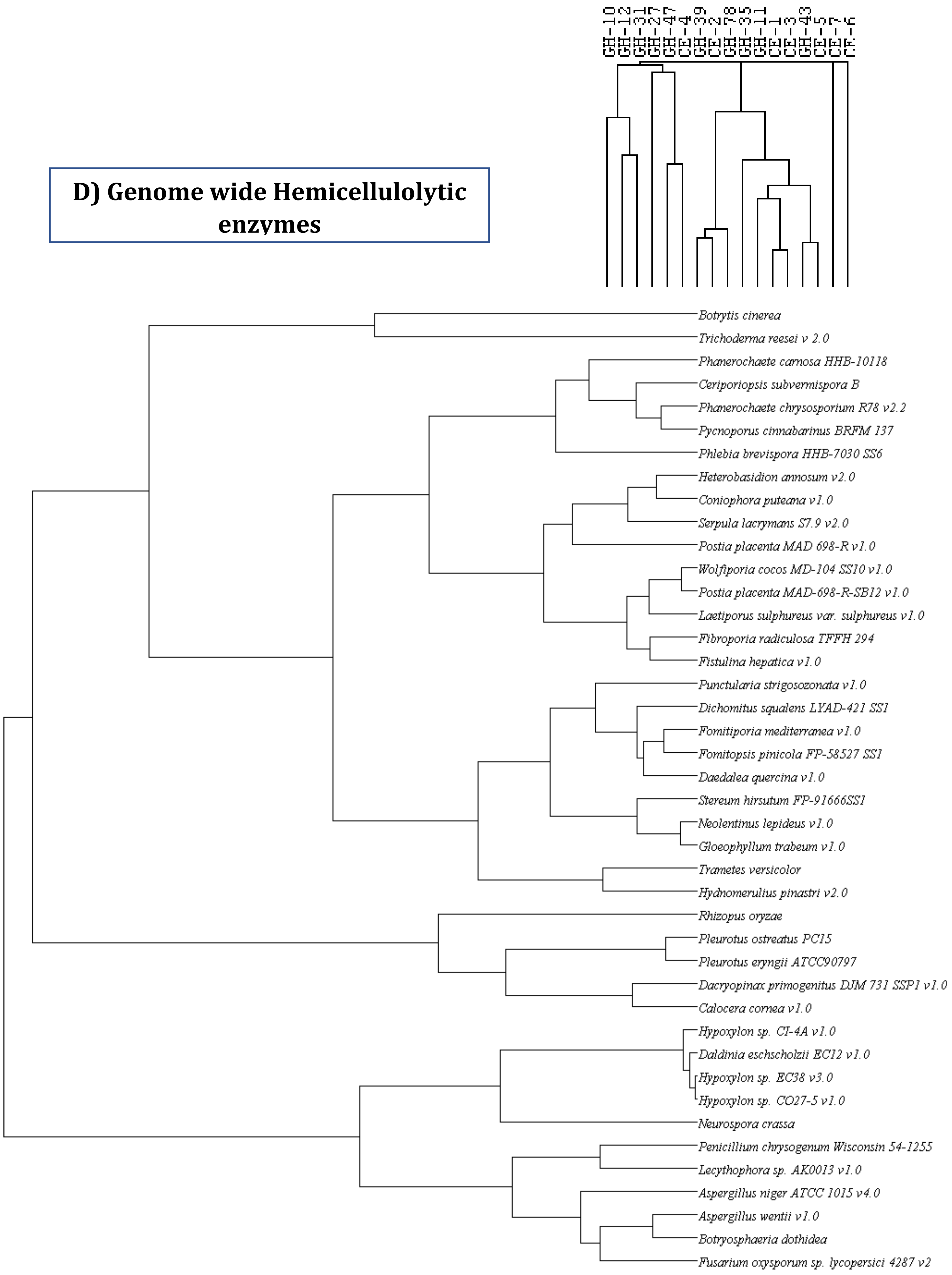

E) Genome wide Pectinolytic enzymes

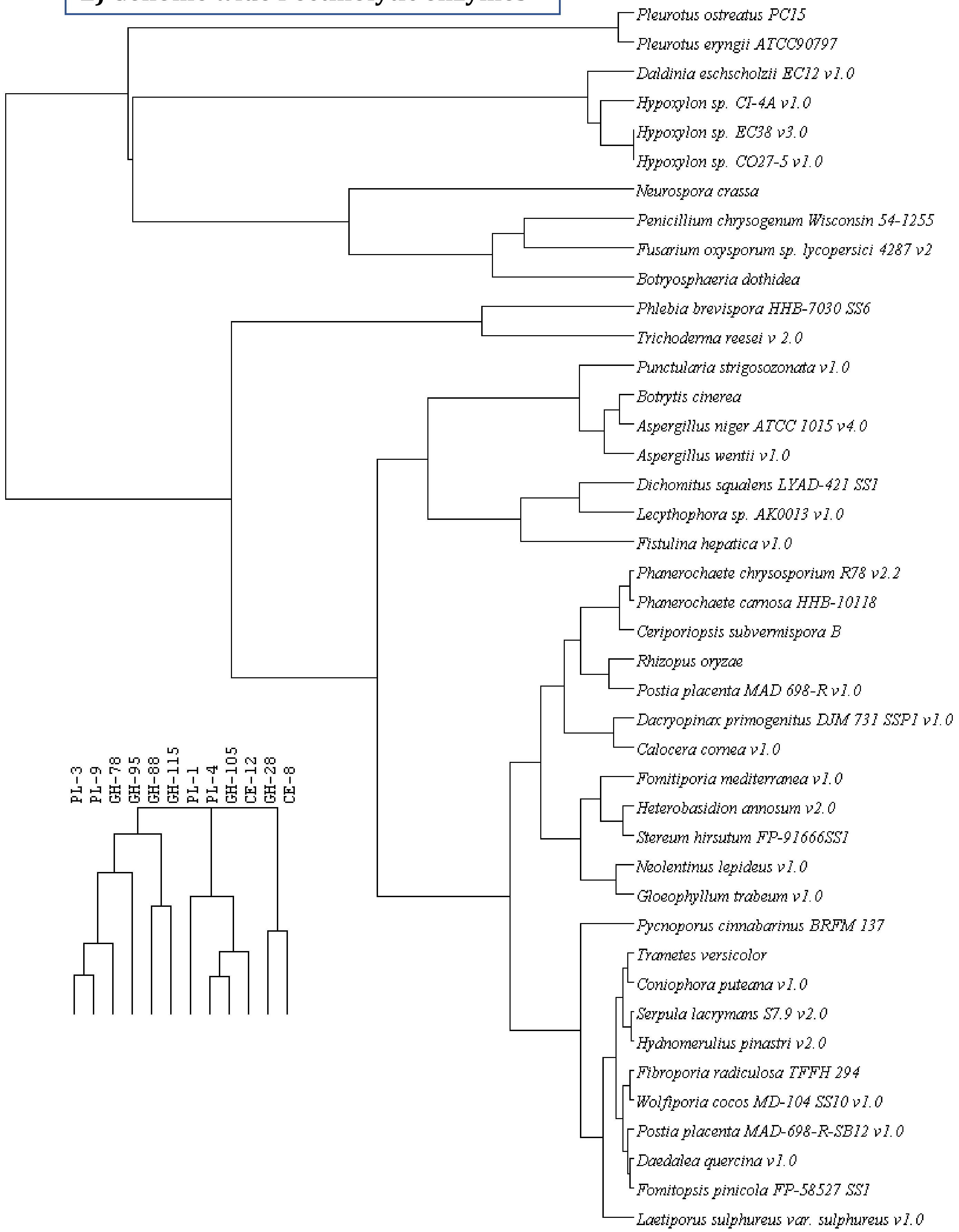

F) Total Lignocellulolytic activities

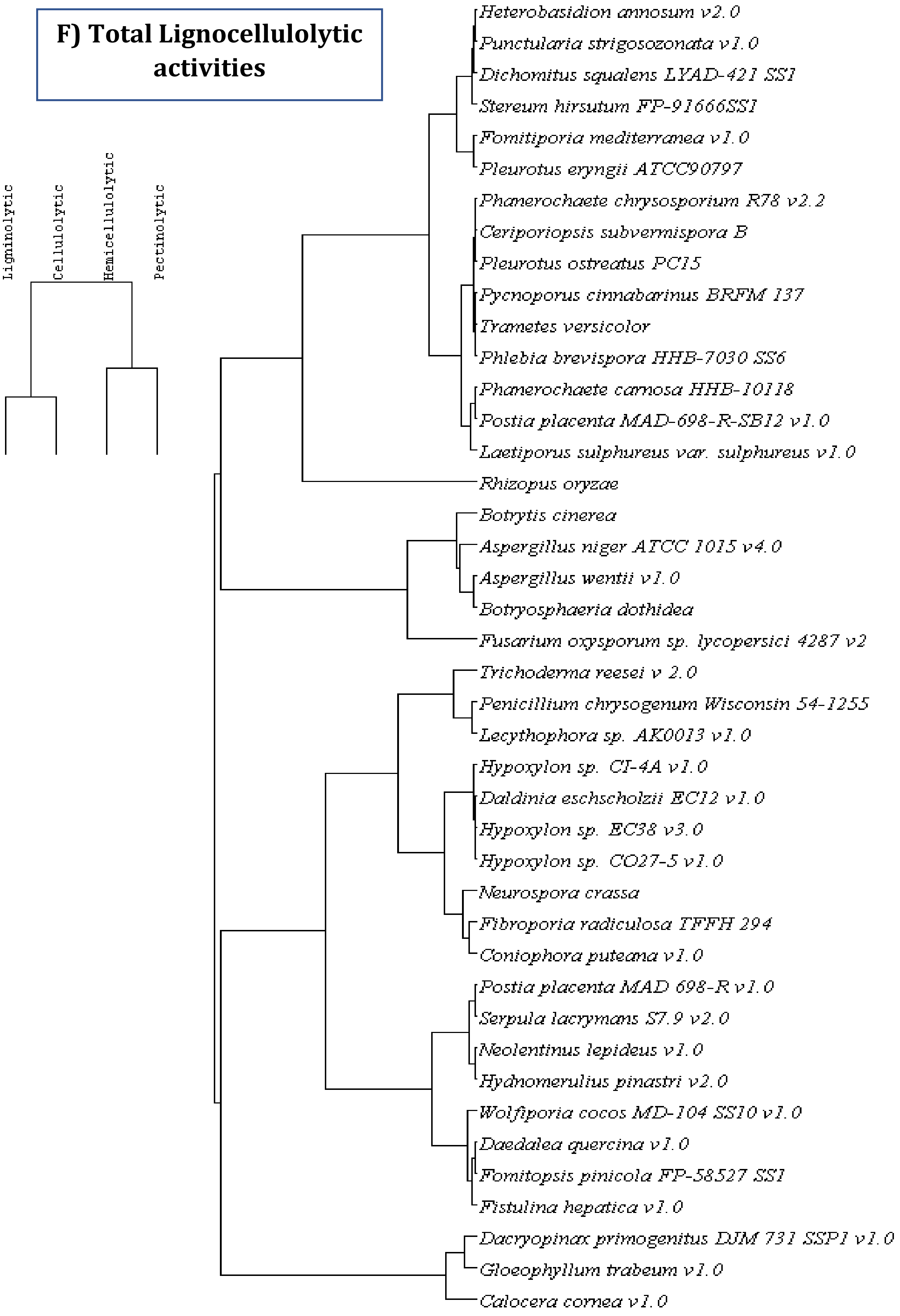

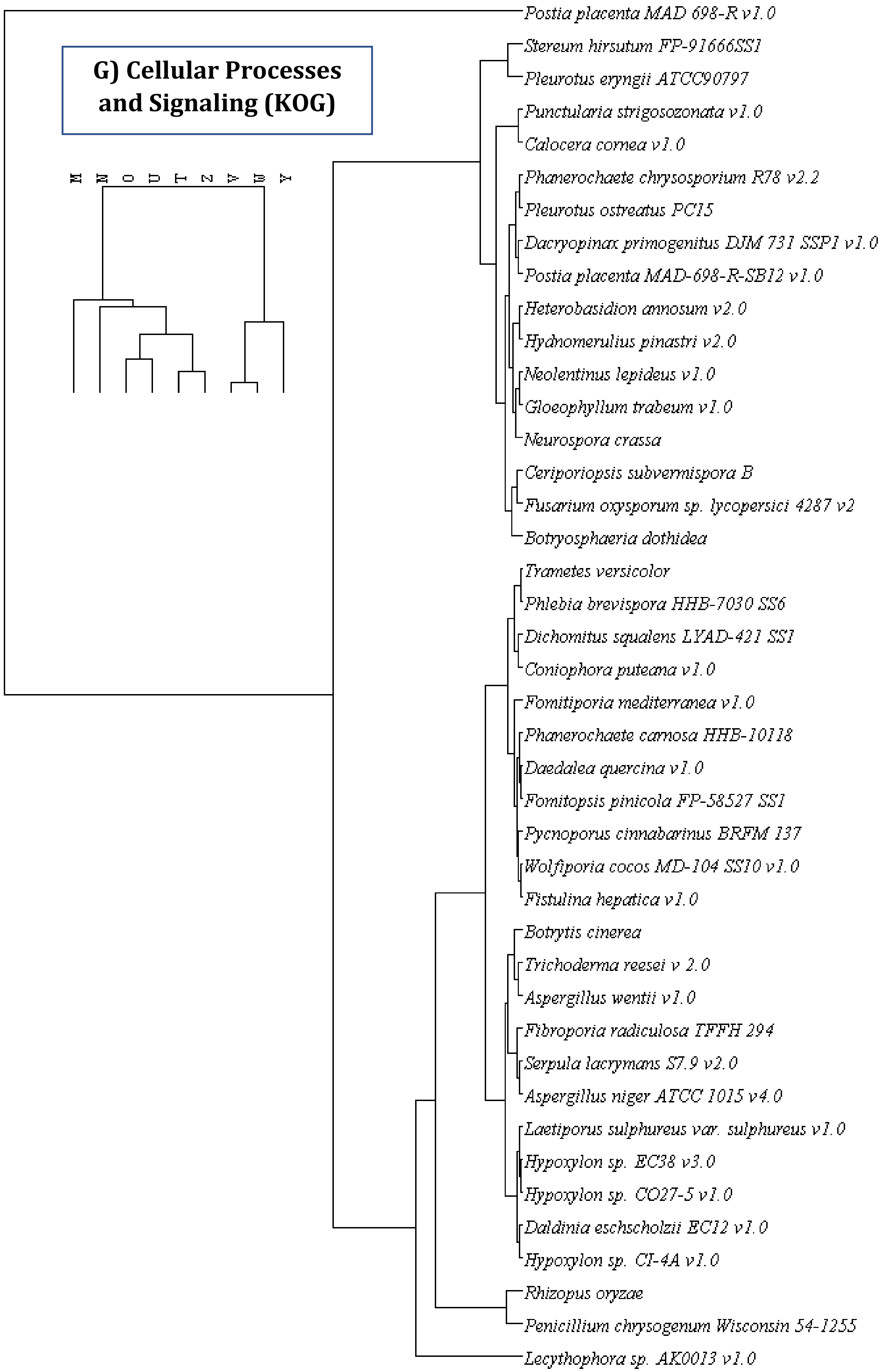
G) Cellular Processes and Signaling (KOG)
Postia placenta MAD 698-R v1.0
Stereum hirsutum FP-91666SS1
Pleurotus eryngii ATCC90797
Punctularia strigosozonata v1.0
Calocera cornea v1.0
Phanerochaete chrysosporium R78 v2.2
Pleurotus ostreatus PC15
Dacryopinax primogenitus DJM 731 SSP1 v1.0
Postia placenta MAD-698-R-SB12 v1.0
Heterobasidion annosum v2.0
Hydnomerulius pinastri v2.0
Neolentinus lepideus v1.0
Gloeophyllum trabeum v1.0
Neurospora crassa
Ceriporiopsis subvermispora B
Fusarium oxysporum sp. lycopersici 4287 v2
Botryosphaeria dothidea
Trametes versicolor
Phlebia brevispora HHB-7030 SS6
Dichomitus squalens LYAD-421 SS1
Coniophora puteana v1.0
Fomitiporia mediterranea v1.0
Phanerochaete carnosa HHB-10118
Daedalea quercina v1.0
Fomitopsis pinicola FP-58527 SS1
Pycnoporus cinnabarinus BRFM 137
Wolfiporia cocos MD-104 SS10 v1.0
Fistulina hepatica v1.0
Botrytis cinerea
Trichoderma reesei v 2.0
Aspergillus wentii v1.0
Fibroporia radiculosa TFFH 294
Serpula lacrymans S7.9 v2.0
Aspergillus niger ATCC 1015 v4.0
Laetiporus sulphureus var. sulphureus v1.0
Hypoxylon sp. EC38 v3.0
Hypoxylon sp. CO27-5 v1.0
Daldinia eschscholzii EC12 v1.0
Hypoxylon sp. CI-4A v1.0
Rhizopus oryzae
Penicillium chrysogenum Wisconsin 54-1255
Lecythophora sp. AK0013 v1.0

H) Information Storage Processing (KOG)

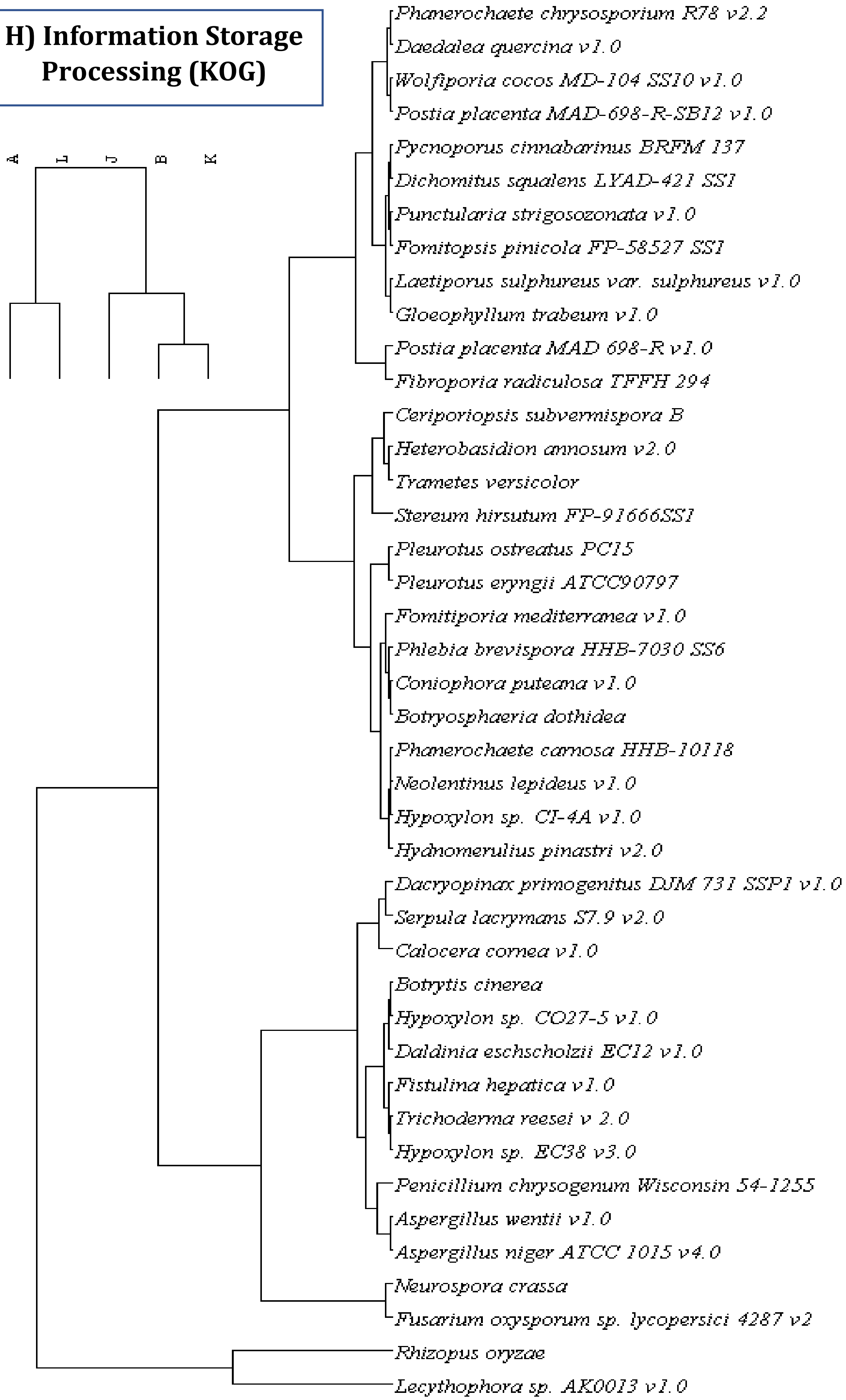

I) Metabolism (KOG)

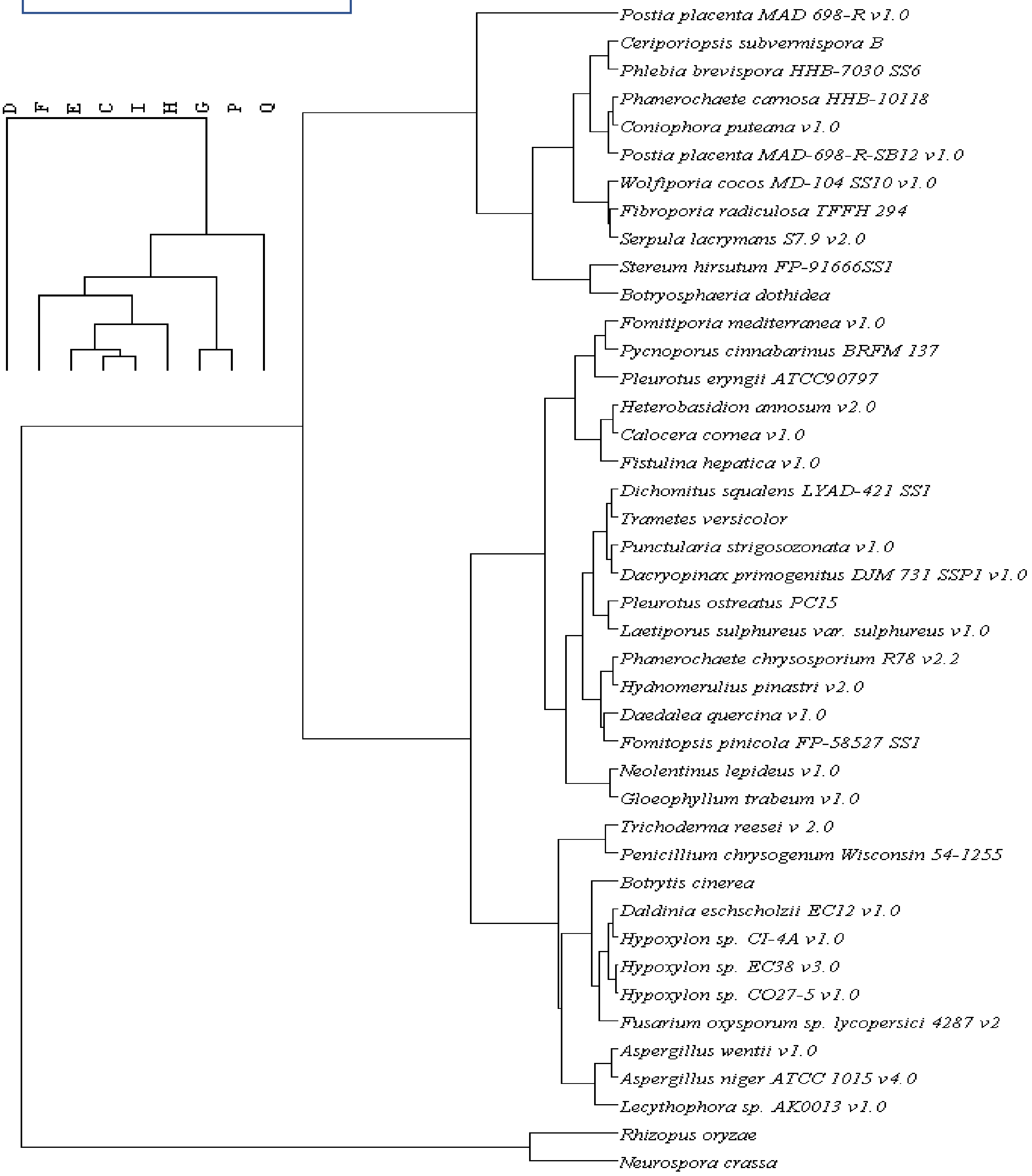

A

Class	P.ch	P.ca	C.su	H.an	F.me	P.cin	D.sq	T.ve	P.st	P.br	B.ci	P.os	S.hi	P.er
AA1_1	0	0	7	12	10	5	11	7	12	8	10	11	15	10
AA1_2	1	1	1	1	1	1	1	2	1	2	1	1	2	1
AA1_3	4	10	1	3	1	1	1	1	0	1	2	0	2	0
AA2	15	11	18	9	17	11	12	26	11	15	0	9	6	8
AA3_1	1	1	1	1	1	1	1	1	1	1	4	1	1	1
AA3_2	34	32	17	29	23	20	27	17	18	32	23	36	40	34
AA3_3	3	4	4	3	3	2	4	4	4	6	2	4	7	10
AA3_4	1	0	0	0	0	2	0	1	1	1	0	0	0	0
AA4	0	0	0	0	0	0	0	0	0	0	1	0	0	0
AA5_1	7	6	3	5	4	7	9	9	9	8	3	16	8	14
AA5_2	0	0	0	0	0	0	0	0	0	0	3	0	0	0
AA6	4	3	0	1	3	1	1	1	2	4	1	2	1	2
AA8	2	2	2	2	1	2	2	2	1	2	7	1	2	1
AA9	16	11	9	10	13	17	15	18	14	12	10	29	16	31
AA11	0	0	0	0	0	0	0	0	0	0	2	0	0	0
AA12	1	3	0	0	0	0	0	0	1	1	0	1	0	1
AA13	0	0	0	0	0	0	0	0	0	0	1	0	0	0

B

Class	P.pl	F.ra	W.co	D.pr	D.qu	L.su	P.pl	N.le	S.la	C.co	G.tr	F.he	F.pi	H.pi	C.pu
AA1_1	4	2	3	0	3	3	2	4	4	0	4	3	5	9	6
AA1_2	1	1	1	2	1	1	1	1	1	2	1	1	1	1	1
AA1_3	1	1	1	3	1	3	1	0	1	3	0	2	1	1	1
AA2	1	1	1	0	1	1	1	0	0	0	0	0	1	0	0
AA3_1	1	0	0	0	0	0	0	0	2	0	1	3	0	1	2
AA3_2	15	16	8	6	13	26	24	23	8	13	0	10	16	9	14
AA3_3	2	6	6	1	4	4	5	3	5	2	2	2	5	4	5
AA3_4	0	0	0	0	0	0	0	0	2	0	1	0	0	2	0
AA4	0	0	0	2	0	0	0	1	0	1	3	0	0	0	0
AA5_1	2	3	4	3	4	5	3	2	3	3	2	3	4	5	6
AA5_2	0	0	0	0	0	0	0	0	0	0	0	0	0	0	0
AA6	0	1	1	1	1	1	1	3	2	1	3	2	1	2	2
AA8	0	0	0	0	0	0	0	0	4	0	0	3	0	4	4
AA9	0	2	2	0	4	2	2	4	5	0	4	7	4	15	10

C

Class	T.re	R.or	A.we	P.ch	D.es	Hypo	A.ni	Hy.EC38	N.cr	Hy.CO27	F.ox	Lecy	B.do
AA1_1	5	0	5	6	2	3	4	3	0	3	3	3	5
AA1_2	2	1	3	2	2	2	3	2	2	2	3	4	3
AA1_3	1	4	3	1	4	4	7	4	8	4	16	5	13
AA2	0	0	1	0	1	2	0	2	0	2	1	2	4
AA3_1	0	1	2	1	4	4	2	4	2	4	5	2	5
AA3_2	10	1	26	19	18	23	26	24	3	24	26	11	36
AA3_3	2	0	1	1	4	6	2	6	1	6	3	2	1
AA3_4	0	0	0	3	3	2	2	3	1	2	1	2	4
AA4	0	0	2	1	3	3	1	3	0	3	1	3	4
AA5_1	1	2	0	0	1	1	0	1	1	1	1	1	2
AA5_2	0	0	1	3	0	0	0	0	1	0	4	0	1
AA6	1	5	2	1	1	1	1	1	1	1	1	1	1
AA8	2	0	4	1	7	6	4	8	7	7	14	8	5
AA9	3	0	8	4	30	27	7	27	14	28	16	22	15
AA10	0	0	0	0	0	0	0	0	0	0	0	0	0
AA11	3	0	6	3	4	4	2	4	4	4	4	5	6
AA12	1	0	0	0	3	3	0	3	3	3	3	5	0
AA13	0	0	0	2	1	1	0	1	1	1	2	1	0

Figure S3: Heatmaps showing the genome wide distribution of ligninolytic enzymes (AA- auxiliary activity) in selected popular white rot, brown rot and soft rot fungi

A

Class	P.ch	P.ca	C.su	H.an	F.me	P.cin	D.sq	T.ve	P.st	P.br	B.ci	P.os	S.hi	P.er
GH-1	2	2	3	2	5	2	4	1	1	2	5	3	3	3
GH-2	2	2	4	3	2	3	4	5	4	2	2	3	3	4
GH-3	10	11	6	11	8	8	8	13	14	8	16	13	17	13
GH-5	19	24	18	16	20	20	19	22	18	23	16	21	20	20
GH-6	1	1	0	1	2	1	1	1	1	1	1	3	1	3
GH-7	8	5	3	1	2	3	4	4	5	4	3	16	3	17
GH-8	0	0	0	0	0	0	0	0	0	0	0	0	0	0
GH-9	1	1	0	1	1	1	0	1	1	1	0	1	1	1
GH-44	0	0	0	0	0	0	1	0	1	2	0	1	1	1
GH-45	2	1	2	2	1	2	1	2	1	3	2	3	1	3
GH-48	0	0	0	0	0	0	0	0	0	0	0	0	0	0
GH-74	4	2	1	1	4	1	1	1	2	1	0	3	2	3
GH-38	1	3	1	1	1	1	1	1	1	1	1	1	1	1

B

Class	P.pl	F.ra	W.co	D.pr	D.qu	L.su	P.pl	N.le	S.la	C.co	G.tr	F.he	F.pi	H.pi	C.pu
GH-1	2	2	1	1	2	2	2	2	3	1	5	1	2	3	3
GH-2	5	3	3	3	3	4	4	3	3	1	4	1	4	3	5
GH-3	8	7	8	9	8	7	6	9	10	11	11	14	11	13	13
GH-5	12	15	18	24	18	20	17	23	21	23	19	15	19	23	21
GH-6	0	0	0	0	0	0	0	0	1	0	0	0	0	1	2
GH-7	0	0	0	0	0	2	0	0	0	0	0	4	0	4	2
GH-8	0	0	0	0	0	0	0	0	0	0	0	0	0	0	0
GH-9	0	0	0	1	0	0	0	1	1	1	1	0	0	1	1
GH-44	0	0	0	0	0	0	0	0	0	0	0	0	0	0	0
GH-45	1	1	0	1	1	2	1	2	0	2	1	2	2	1	1
GH-48	0	0	0	0	0	0	0	0	0	0	0	0	0	0	0
GH-74	0	0	0	0	0	0	0	1	1	0	1	1	0	1	0
GH-38	1	1	1	1	1	1	1	1	1	1	1	1	1	1	1

C

Class	T.re	R.or	A.we	P.ch	D.es	Hypo	A.ni	Hy.EC38	N.cr	Hy.CO27	F.ox	Lecy	B.do
GH-1	2	0	3	3	1	2	3	2	2	2	6	3	6
GH-2	8	0	5	7	9	8	6	10	6	10	9	6	5
GH-3	13	6	16	16	18	21	19	22	11	21	33	17	22
GH-5	8	7	11	14	15	15	10	18	6	17	21	19	19
GH-6	1	0	1	1	3	2	2	2	3	2	1	3	1
GH-7	2	0	3	2	5	4	2	5	5	5	3	9	2
GH-8	0	1	0	0	0	0	0	0	0	0	0	0	0
GH-9	0	5	0	0	0	0	0	0	0	0	0	0	0
GH-44	0	0	0	0	0	0	0	0	0	0	0	0	0
GH-45	1	5	0	0	1	1	0	1	1	1	1	4	4
GH-48	0	0	0	0	0	0	0	0	0	0	0	0	0
GH-74	1	0	0	0	1	1	1	1	1	1	1	1	0
GH-38	1	2	1	1	1	1	1	1	1	1	1	1	1

Figure S4: Heatmaps showing the genome wide distribution of cellulolytic enzymes (GH-glycosidic hydrolases) in selected popular white rot, brown rot and soft rot fungi.

A

Class	P.ch	P.ca	C.su	H.an	F.me	P.cin	D.sq	T.ve	P.st	P.br	B.ci	P.os	S.hi	P.er
GH-10	6	5	6	2	4	6	5	6	5	8	2	3	6	4
GH-11	1	1	1	0	0	0	0	0	1	0	2	2	1	2
GH-39	0	0	0	0	0	0	0	0	0	0	1	0	0	0
GH-12	2	3	2	4	3	3	3	5	2	2	4	2	5	2
GH-43	4	4	2	4	7	3	7	3	7	2	6	8	12	7
GH-47	5	5	4	6	5	5	5	5	5	9	9	9	6	10
GH-27	3	3	4	4	4	2	6	4	5	2	4	7	5	8
GH-31	6	8	5	9	5	5	6	5	8	5	5	8	8	5
GH-35	3	4	1	4	2	2	3	2	4	4	4	4	7	4
GH-78	1	1	1	2	2	2	5	3	7	1	7	2	3	4
CE-1	4	2	2	1	0	3	0	3	2	1	2	2	1	2
CE-2	0	0	0	0	0	0	0	0	0	0	0	0	0	0
CE-3	0	0	0	0	0	0	0	0	0	0	1	0	0	0
CE-4	4	2	3	5	4	3	2	3	5	2	5	11	7	10
CE-5	0	0	0	0	0	0	0	0	1	1	11	0	1	0
CE-6	0	0	0	0	0	0	0	0	0	0	0	0	0	0
CE-7	0	0	0	0	0	0	0	0	0	0	0	0	0	0

B

Class	P.pl	F.ra	W.co	D.pr	D.qu	L.su	P.pl	N.le	S.la	C.co	G.tr	F.he	F.pi	H.pi	C.pu
GH-10	1	3	4	3	4	3	3	2	1	2	3	2	3	3	3
GH-11	0	0	0	0	0	0	0	0	0	2	0	0	0	0	0
GH-39	0	0	0	0	0	0	0	0	0	0	0	0	0	0	0
GH-12	2	2	2	1	2	2	2	2	2	1	2	2	2	3	4
GH-43	0	3	1	5	4	1	1	5	2	4	6	2	7	3	6
GH-47	7	6	5	4	5	5	5	4	6	4	4	7	6	5	9
GH-27	2	3	3	2	4	3	3	3	3	2	3	4	4	6	4
GH-31	6	6	5	6	4	4	4	5	5	8	5	6	5	6	12
GH-35	2	1	2	1	1	1	1	2	3	1	2	3	2	2	2
GH-78	1	3	3	0	3	3	3	1	2	0	2	5	4	2	2
CE-1	0	0	0	0	1	2	0	0	0	0	1	0	0	2	0
CE-2	0	0	0	0	0	0	0	0	0	0	0	0	0	0	0
CE-3	0	0	0	0	0	0	0	0	0	0	0	0	0	0	0
CE-4	2	3	4	11	3	3	3	5	6	7	5	4	3	3	9
CE-5	0	0	0	0	0	0	0	0	0	0	0	0	0	0	1
CE-6	0	0	0	0	0	0	0	0	0	0	0	0	0	0	0
CE-7	0	0	0	0	0	0	0	0	0	0	0	0	0	0	0

C

Class	T.re	R.or	A.we	P.ch	D.es	Hypo	A.ni	Hy.EC38	N.cr	Hy.CO27	F.ox	Lecy	B.do
GH-10	1	0	4	3	3	2	2	3	4	3	5	6	6
GH-11	3	0	1	1	3	2	3	2	2	2	3	5	1
GH-39	1	0	0	2	0	0	0	0	0	0	4	0	2
GH-12	2	0	3	4	0	1	4	0	2	0	4	2	4
GH-43	2	2	14	13	20	19	11	21	7	22	35	19	23
GH-47	8	11	6	5	9	7	6	9	9	9	10	11	11
GH-27	8	2	2	2	3	1	5	3	0	2	4	3	7
GH-31	4	3	10	12	5	6	7	6	6	6	10	14	11
GH-35	1	1	4	4	5	5	5	4	2	4	7	3	4
GH-78	1	0	5	5	2	3	9	3	2	3	18	5	8
CE-1	2	0	2	2	7	7	2	8	6	8	7	9	4
CE-2	0	0	0	0	0	0	0	0	0	0	1	0	0
CE-3	3	0	2	3	9	9	0	8	3	8	6	4	0
CE-4	3	35	5	4	6	6	4	6	5	6	9	6	7
CE-5	4	0	8	4	6	5	5	7	3	7	12	8	16
CE-6	0	0	0	0	0	0	0	0	0	0	0	0	0
CE-7	0	0	0	0	0	0	0	0	0	0	0	0	0

Figure S5: Heatmaps showing the genome wide distribution of hemicellulolytic enzymes (GH-glycosidic hydrolases and CE-Carbohydrate esterases) in selected popular white rot, brown rot and soft rot fungi

A

Class	P.ch	P.ca	C.su	H.an	F.me	P.cin	D.sq	T.ve	P.st	P.br	B.ci	P.os	S.hi	P.er
PL-1	0	0	0	2	2	0	0	0	4	0	7	10	4	19
PL-3	0	0	0	0	0	0	0	0	0	0	2	3	0	3
PL-4	0	0	0	1	0	1	1	1	3	1	0	2	3	2
PL-9	0	0	0	0	0	0	0	0	0	0	0	0	0	0
GH-28	5	4	6	8	17	7	7	11	13	5	19	6	17	8
GH-78	1	1	1	2	2	2	5	3	7	1	7	2	3	4
GH-88	1	1	1	1	2	2	1	1	1	1	1	1	1	2
GH-95	1	1	1	1	2	1	1	1	1	3	2	1	1	1
GH-105	0	0	0	2	1	0	1	0	2	0	2	2	2	2
GH-115	1	1	2	1	3	2	2	2	1	2	1	1	2	1
CE-8	2	2	2	3	3	2	3	2	6	3	5	2	5	2
CE-12	0	0	0	2	2	2	2	0	1	0	3	2	3	2

B

Class	P.pl	F.ra	W.co	D.pr	D.qu	L.su	P.pl	N.le	S.la	C.co	G.tr	F.he	F.pi	H.pi	C.pu
PL-1	0	0	0	0	0	0	0	0	0	0	0	1	0	0	0
PL-3	0	0	0	0	0	0	0	0	0	0	0	0	0	0	0
PL-4	0	0	0	0	0	0	0	0	0	0	2	1	0	0	0
PL-9	0	0	0	0	0	0	0	0	0	0	0	0	0	0	0
GH-28	7	9	9	6	9	8	8	10	7	7	10	6	12	9	13
GH-78	1	3	3	0	3	3	3	1	2	0	2	5	4	2	2
GH-88	1	1	1	1	1	1	1	1	1	1	1	1	1	1	1
GH-95	1	1	1	0	1	1	1	1	1	0	1	1	1	1	1
GH-105	0	0	0	0	0	0	0	2	0	1	2	3	1	0	0
GH-115	0	2	2	2	1	3	1	1	1	2	2	4	1	1	2
CE-8	4	2	1	3	1	1	2	2	2	2	2	2	2	2	2
CE-12	0	0	0	0	0	0	0	0	0	0	0	0	0	0	0

C

Class	T.re	R.or	A.we	P.ch	D.es	Hypo	A.ni	Hy.EC38	N.cr	Hy.CO27	F.ox	Lecy	B.do
PL-1	0	0	5	5	2	3	7	2	1	2	10	2	8
PL-3	0	0	2	1	0	1	0	1	1	1	7	1	9
PL-4	0	0	3	3	5	5	2	5	1	5	3	3	5
PL-9	0	0	0	0	0	0	0	0	0	0	2	0	1
GH-28	4	18	12	5	3	4	21	3	2	3	15	6	10
GH-78	1	0	5	5	2	3	9	3	2	3	18	5	8
GH-88	0	0	0	0	1	0	1	0	0	0	3	1	1
GH-95	4	0	1	1	0	0	2	0	0	0	2	1	3
GH-105	1	0	2	2	3	4	2	3	2	3	4	2	4
GH-115	1	0	1	0	1	1	0	1	1	1	3	3	2
CE-8	0	6	2	2	1	1	3	2	2	2	8	3	3
CE-12	0	0	2	2	2	2	2	2	1	2	4	3	4

Figure S6: Heatmaps showing the genome wide distribution of pectinolytic enzymes (GH-glycosidic hydrolases, PL-polysaccharide lyases and CE-Carbohydrate esterases) in selected popular white rot, brown rot and soft rot fungi
